# Understanding the Mechanisms Behind the Response to Environmental Perturbation in Microbial Mats: A Metagenomic-Network Based Approach

**DOI:** 10.3389/fmicb.2018.02606

**Published:** 2018-11-28

**Authors:** Valerie De Anda, Icoquih Zapata-Peñasco, Jazmín Blaz, Augusto Cesar Poot-Hernández, Bruno Contreras-Moreira, Marcos González-Laffitte, Niza Gámez-Tamariz, Maribel Hernández-Rosales, Luis E. Eguiarte, Valeria Souza

**Affiliations:** ^1^Departamento de Ecología Evolutiva, Instituto de Ecología, Universidad Nacional Autónoma de México, Ciudad de México, Mexico; ^2^Dirección de Investigación en Transformación de Hidrocarburos, Instituto Mexicano del Petróleo, Eje Central Lázaro Cárdenas, Ciudad de México, Mexico; ^3^Laboratorio Nacional de Ciencias de la Sostenibilidad, Instituto de Ecología, Universidad Nacional Autónoma de México, Ciudad de México, Mexico; ^4^Departamento de Ingeniería de Sistemas Computacionales y Automatización, Instituto de Investigaciones en Matemáticas Aplicadas y en Sistemas, UNAM, Ciudad Universitaria, Ciudad de México, Mexico; ^5^Estación Experimental de Aula Dei, Consejo Superior de Investigaciones Científicas, Zaragoza, Spain; ^6^Fundación ARAID, Zaragoza, Spain; ^7^Instituto de Matemáticas, UNAM Juriquilla, Juriquilla, Mexico

**Keywords:** time series ecological networks, environmental perturbation, MEBS, microbial mats, rare biosphere, network motifs

## Abstract

To date, it remains unclear how anthropogenic perturbations influence the dynamics of microbial communities, what general patterns arise in response to disturbance, and whether it is possible to predict them. Here, we suggest the use of microbial mats as a model of study to reveal patterns that can illuminate the ecological processes underlying microbial dynamics in response to stress. We traced the responses to anthropogenic perturbation caused by water depletion in microbial mats from Cuatro Cienegas Basin (CCB), Mexico, by using a time-series spatially resolved analysis in a novel combination of three computational approaches. First, we implemented MEBS (Multi-genomic Entropy-Based Score) to evaluate the dynamics of major biogeochemical cycles across spatio-temporal scales with a single informative value. Second, we used robust Time Series-Ecological Networks (TS-ENs) to evaluate the total percentage of interactions at different taxonomic levels. Lastly, we utilized network motifs to characterize specific interaction patterns. Our results indicate that microbial mats from CCB contain an enormous taxonomic diversity with at least 100 phyla, mainly represented by members of the rare biosphere (RB). Statistical ecological analyses point out a clear involvement of anaerobic guilds related to sulfur and methane cycles during wet versus dry conditions, where we find an increase in fungi, photosynthetic, and halotolerant taxa. TS-ENs indicate that in wet conditions, there was an equilibrium between cooperation and competition (positive and negative relationships, respectively), while under dry conditions there is an over-representation of negative relationships. Furthermore, most of the keystone taxa of the TS-ENs at family level are members of the RB and the microbial mat core highlighting their crucial role within the community. Our results indicate that microbial mats are more robust to perturbation due to redundant functions that are likely shared among community members in the highly connected TS-ENs with density values close to one (≈0.9). Finally, we provide evidence that suggests that a large taxonomic diversity where all community members interact with each other (low modularity), the presence of permanent of low-abundant taxa, and an increase in competition can be potential buffers against environmental disturbance in microbial mats.

## Introduction

Understanding the responses and the mechanisms that constrain and promote microbial adaptation in face of environmental perturbation is crucial to evaluate the ecosystem impact at global scales. Microbial ecological studies have demonstrated that diverse microbial communities tend to be more stable over time by promoting functional redundancy, whereas after a disturbance the community richness and diversity tends to decline (Girvan et al., 2005; Gihring et al., 2011; Hunting et al., 2015). Recent studies suggest that the probability that the community will return to its previous state following a small perturbation (here after referred as microbial stability, see Allesina et al., 2015; Borrelli et al., 2015; Grilli et al., 2016) could be related to the great genetic reservoir of low abundant taxa, also known as the rare biosphere (RB), and the microbial interactions that could have a crucial role providing a buffer against environmental disturbances influencing both community assembly and stability (Hunt and Ward, 2015; Konopka et al., 2015; Lynch and Neufeld, 2015; Jousset et al., 2017; Karpinets et al., 2018; Rivett and Bell, 2018). Yet, to date it is unclear how microbial community dynamics (i.e., composition and relationships) are influenced by environmental constraints, which largely shape the degree of resistance, resilience, and functional redundancy of a microbial community (Allison and Martiny, 2008; Fuhrman, 2009; Newton et al., 2011; Bissett et al., 2013; Konopka et al., 2015).

Natural microbial communities are highly complex systems that are in constant flux through spatial and temporal scales, where even minor perturbation can significantly reorder the function of each community member and the interaction network (Konopka et al., 2015). In this study we focused on community level patterns in stable microbial communities during environmental perturbation and the possible mechanisms that facilitates or disrupts microbial community stability and their ability to adapt to change. Due to their capacity to perform most of the biogeochemical cycles in a physically and chemically reduced environment, microbial mats are excellent models of study. Microbial mats are successful ecological communities which have adapted continuously to environmental changes since the Archean Eon (van Gemerden, 1993; Guerrero et al., 2002; Bolhuis et al., 2014; Preisner et al., 2016). Here, we seek to identify the general patterns caused by environmental perturbation. If microbial mats are resilient and resistant over time, we expect that samples taken through time in fixed points in space would present similar community patterns regardless of the disturbance. In contrast, if these communities survived by a constant species turnover, we expect to see systematic differences reflected in the community composition, function, structure, and overall relationships.

Despite their importance, low abundance taxa have been routinely removed from microbial ecology studies (Jousset et al., 2017). Most existing studies using network inference focus on evaluating a small percentage of the strongest interaction pairs, or infer microbial relationships at one single taxonomic level or a few marker genes involved in several metabolic process ignoring if those genes are differentially abundant (Shaw et al., 2017).

In order to comprehensively evaluate complex microbial community dynamics under environmental disturbance, we suggest the implementation of three approaches to complement the standard taxonomic ecological analysis. (1) The analysis of robust time series ecological networks (TS-ENs) at different taxonomic level to access general patterns of microbial relationships. (2) The application of MEBS (Multigenomic Entropy Based Score) to capture the enrichment of biogeochemical cycles or other complex metabolic pathways (De Anda et al., 2017) to provide a quantifiable measure of community response to environmental perturbation. (3) The use of network motifs or building blocks of biological networks that have been applied to the study of development, regulatory, and neuronal networks (Shen-Orr et al., 2002; Prill et al., 2005; Tran et al., 2013), and in ecological food webs of plants and animals (Stouffer et al., 2007; Borrelli et al., 2015; Baiser et al., 2016). The latter is needed since network motifs studies offer the opportunity to bridge the gap between the dynamics of simple modules and the analysis of topological metrics describing the community as a whole (Delmas et al., 2018).

To examine the community response against anthropogenic perturbations caused by water depletion, we studied microbial mats at three sites in the Churince Lagoon in Cuatro Cienegas Basin (CCB) México during 2012–2014. CCB is an extremely oligotrophic oasis characterized by low P concentrations (PO_4_^3-^ as low as 0.1 μM) but relatively high concentrations of inorganic N and thus high N: P ratios (>200:1 by atoms) (Lee et al., 2015, 2017). Paradoxically, despite this nutrient limitation, CCB is a World Wildlife Fund (WWF) hotspot for biodiversity and a wetland of international importance under the RAMSAR convention and is a singularity biodiversity that persisted through time (Souza et al., 2018). However, despite the importance of this site, increasing demand for water by agricultural development (mainly for forage and feed for livestock) has generated critical conservation issues related to the drying of different aquatic systems in the basin. In particular, the desiccation process of the Churince Lagoon, a wetland with a rich input of deep water with magmatic influence (Wolaver et al., 2012) and a high, endemic biodiversity that now is in danger of disappearing (Souza et al., 2007, 2018; De Anda et al., 2018).

In order to focus on the dynamic response of these communities in the face of anthropogenic perturbation, we seek to address the following questions: How are the networks assembled from their basic building blocks? Can we detect the keystone taxa? Is it possible to discriminate between intrinsic community changes and those given by the environmental degradation? Finally, can we mechanistically predict the overall behavior in the community under environmental stress using network inference? To this end, we used high throughput Illumina sequencing to assess the community composition and function as well as the change in the structure and membership networks between sites during and after the anthropogenic perturbation. Our results indicate that even though microbial mats are resilient and resistant taxonomically and metabolically, their network of interactions change during the dry period. During this time, negative interactions predominate under stress while interactions are more balanced in wet conditions.

## Materials and Methods

### Sample Collection and Processing

**FIGURE 1:**
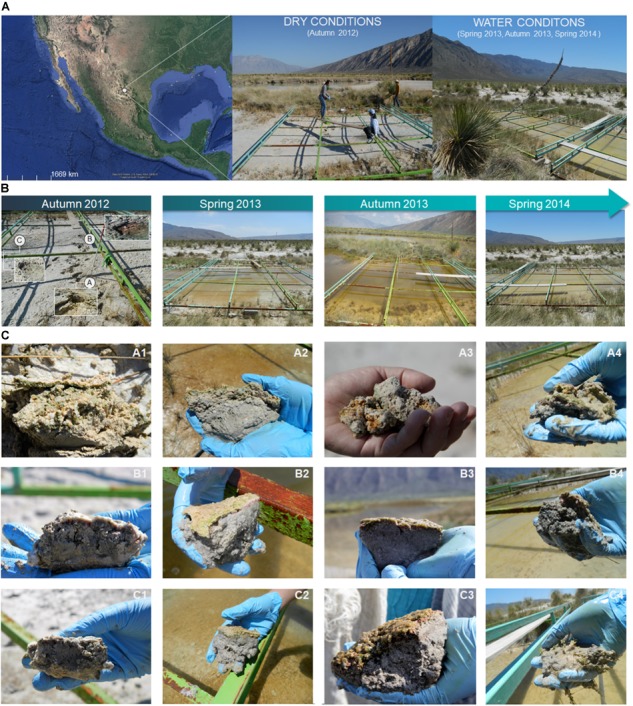
Sampling site and microbial mats samples description. **(A)** The Lagunita pond is located at Cuatro Cienegas Basin (CCB) in state of Coahuila, Mexico Map. Google Maps. Google, 15 Jun 2018. Web, 15 Jun 2018. Here is shown two contrasting conditions during the study period (dry and wet or “water” conditions). **(B)** Specific characteristics of the Lagunita pond are showed during the sampling period ranging from Autumn 2012 (time 1), to Spring 2014 (time 4). **(C)** Microbial mats were sampled seasonally from three geographically separated sites **(A–C)** during two-years resulting in a total of 12 samples (3 sites, 4 time points).

Microbial mats were sampled from a small (ca. 12 m × 4 m) pond named “Lagunita” that is part of the main Churince lake in CCB (26.84810° N, -102.14160° W). Under normal conditions Lagunita pond is shallow with variable water levels (<0.42 cm) (Lee et al., 2015, 2017; Figure 1A). However, during initial fieldwork, we found that long term water extraction for agriculture had finally overturned the water levels and Lagunita pond was almost dry, with a majority of its sediment exposed and in direct contact with the atmosphere, consequently desiccating and displaying green and yellow tonalities. With the exception of two wet patches, that were both covered with a thick white surface with a sulfide smell. We sampled microbial mats from one dry area (Site A: 26.848120°N, -102.141604°W) and two wet patches (Site B: 26.848093°N, -102.141608°W and C: 26.848084°N, -102.141577°W), all less than 3 meters from each other. We sampled seasonally (Autumn and Spring) from 2012 to 2014 (Figure 1B) resulting in a total of 12 samples (Figure 1C).

Triplicate samples of each mat were obtained for each time point (5 cm × 5 cm × 5 cm), using sterile Falcon tubes (50 mL) and then stored at 4°C and subsequently frozen in liquid nitrogen until processing in the lab. Physicochemical water parameters were registered at each sampling time using Hydrolab Mini Sonde 5 Multiprobe SE, with the exception of the initial (dry) sampling (as there was not water to measure). We took great care in preserving the mat structure and, even if we could not split the layers due to the mat consistency, we represented in our DNA isolation all the layers evenly, starting with the upper photic layer and ending with a similar sized black anoxic layer.

### DNA Extractions

Approximately 0.5 g of microbial mats sample was extracted for each replicated sample according to Purdy (2005). Replicate samples for the same site and time point were pooled into a single sample, yielding a total of 12 DNA samples, each one with more than 5 μg of high molecular weight DNA.

### Metagenomic Library Preparation, Sequencing and Quality Assessment

Metagenomic shotgun sequencing libraries were prepared and sequencing at CINVESTAV-LANGEBIO, Irapuato, Mexico. For each sample ∼5 μg of genomic DNA (DO 260/280 ≤1.8) was used with Illumina’s TruSeq nano for library preparation, which supports shearing by Covaris ultrasonication. Fragments were selected according to agarose gel 0.7% at 70 mV in order to obtain an average insert size of 550 bp. Libraries were sequenced using the Illumina MiSeq Paired-End 2 × 300 bp technology with a run in a single plate generating 12Gb of sequence data for all 12 samples. Quality of raw reads was analyzed using FastQC v0.11 (Andrews, 2010). TruSeq Indexed Adapter and barcodes were removed using cutadapt v1.12 (Martin, 2011). Low quality sequences were discarded with Trimmomatic using a sliding window of 4 bp, an average quality per base of 20, and min read length of 36 bp (Bolger et al., 2014). The assembly of the trimmed reads was conducted with Megahit v1.1.1-2 using the option – presets meta-large (Li et al., 2014).

The coding regions were searched from the obtained contigs using Prodigal v2.6.3 (Hyatt et al., 2010) with the -a option, to obtain the translated amino acid sequences of the predicted coding regions and -p meta option. The peptide amino acid sequences were then scanned against Pfam-A v30 (Finn et al., 2015). The abundance profile of each Pfam domain in the metagenomic samples was obtained from a Perl script extract_pfam.pl which is part of the MEBS software suite (De Anda et al., 2017). The resulting FASTA files of sequence contigs have been deposited in the MG-RAST repository under project number mgp80319.

### Taxonomy Assignment

We used the k-mer based taxonomic classification algorithm of One Codex (Minot et al., 2015) to assign microbial taxonomy of the Megahit derived contigs. Briefly, One Codex classifies unknown nucleotide sequences according to the set of signature sequences that are unique to a specific taxonomic group using oligonucleotides of 31 bp (*k* = 31). The taxonomic profiles obtained from the reference-based approach were used for downstream analyses described below.

### Microbial Mats Diversity, Structure and Statistics

Several descriptors of alpha diversity were obtained from Phyloseq-estimate_richness function (McMurdie and Holmes, 2013) implemented in R (R Development Core Team, 2011). In order to estimate the sampling effort, rarefaction curves were obtained for each sampling site using the rarefaction function implemented in Vegan Library in R. Several statistical analyses were performed in order to test for differences between samples and estimate components of variation due to year, site, or water conditions. We first performed a permutational multivariate analysis of variance (PERMANOVA) using R-vegan function Adonis in order to establish the differences between sites and times. A pairwise comparison among samples for the same site and water conditions was performed using the STAMP v2.1.3 program (Parks et al., 2014) in order to statistically identify the significant differences among genera within each sample by using Welch’s *t*-test type two-sided, with the confidence interval (CI) method of Welch’s inverted adjustment of 0.95. Then, we compared the taxonomic presence/absence profile from the microbial mats under dry condition versus those from the rest of the sampling, the particular and shared taxa across water conditions were plotted with Venn-Euler diagrams using a web-based tool^1^. Finally, we identified the core of microbial mats (those genera present across space and time in all microbial mat samples) using the *parse_pangenome_matrix.pl* script part of the software GET_HOMOLOGUES (Contreras-Moreira and Vinuesa, 2013).

### Biogeochemical Cycling Dynamics

We used MEBS (De Anda et al., 2017) to evaluate the metabolic machinery of C, O, N, S, and Fe cycles in the microbial mats across time by using a single value measured in bits (informational units). Briefly, FASTA peptide sequences for each microbial mat, obtained with Prodigal, are taken as input of the main script *mebs.pl.* We used the -comp option to compute the metabolic completeness of sulfur and methane cycle (currently N and Fe cycles are also supported). Cycles enriched in a given sample are recognized by using the –fdr (False Discovery Rate). In this case we used a restrictive FDR of 0.0001. We performed ROC analysis described in De Anda et al. (2017) for each cycle, computing several cut-offs for fixed FDR rates. In this way, by using restrictive FDR we can control the rate of true positives and false positives obtained in each cycle. The exact values for each cycle are shown in the config file of MEBS github repository^2^. The details of those analyses will be published elsewhere, including the benchmark of each cycle across two-thousand non-redundant genomes.

To contrast the biogeochemical cycles across several environments, we used publicly available metagenomes from MG-RAST of stromatolites from Highborne Cay, Bahamas 4449591.3 4449590.3 (Khodadad and Foster, 2012), Polar Microbial mats 4445126.3 4445129.3 (Varin et al., 2012); freshwater microbial mats from CCB 4442467.3 4442466.3 4441363.3 4441347.3 (Bonilla-Rosso et al., 2012; Peimbert et al., 2012) and stromatolites from CCB, 4440060.4 4440067.3 (Desnues et al., 2008; Breitbart et al., 2009); microbial mats from Yellowstone 4443746.3 4443747.3 4443762.3 4443749.3 4443750.3 (Bhaya et al., 2007); purple sulfur bacteria biofilm (Wilbanks et al., 2014); hydrothermal vents 4487624.3 4487625.3 (Tang et al., 2013), 4449206.3 (Jiménez et al., 2012); microorganisms from the vent-associated polychaete worm *Alvinella pompejana* 4441102.3 (Grzymski et al., 2008); acid mine drainage 4441138.3 4441137.3 (AMD) (Jiao et al., 2011); polar cryoconite 4491734.3; freshwater microbialites from Pavilion Lake, Clinton Creek described in White et al. (2015, 2016); hypersaline microbial mats from marine environments described in Ruvindy et al. (2016) and Guerrero Negro (Kunin et al., 2008).

### Network Inference

We used the time-series Lotka-Volterra-based network inference approach MetaMIS (Shaw et al., 2016) to infer the underlying interactions from microbial mats collected during and after the perturbation event. For each site, the non-normalized taxonomic classifications (ranging from Phylum to Family) were used to compute the consensus networks. Due to the large amount of network interactions generated at lower hierarchical levels and the limitation of computing power (Intel Core i7-4500U CPU @3.20 GHz processor and 16Gb RAM), we were not able to obtain the consensus networks at genera level. Considering that the three studied sites are very close together, we constructed a “high order-network” by concatenating the consensus networks of the three sites. We named it “global network” and it was constructed to find general patterns that could reflect the behavior of the mats within Lagunita pond.

The consensus networks inferred from MetaMIS can include several types of interaction patterns, yet we decided to further separate them into more simple networks displaying only either positive or negative relationships. For each site we obtained three different networks (consensus, positive and negative) resulting in a total of 36 Time-Series Ecological networks (TS-ENs). The global network was also separated by type of interaction at every taxonomic level, resulting in 12 global TS-ENs per sampling time. In total, we obtained 48 TS-ENs in our study.

### Motif Discovery

Network motifs are defined as a set of recurring circuits on *n* nodes. Nodes can represent biological entities such as OTUs, species, genes or proteins. Network motifs are patterns of interactions from which the networks are built. These patterns occur in complex networks more often than expected in a random network (Milo, 2002; Alon, 2007; Baiser et al., 2016). In order to compare our data with those derived from the ecological theory developed in food webs and the tractable number of network motifs, we focused only on the 13 possible 3-node network motifs. For comparison, there are 199 and 9364 motifs for four and five node subgraphs, respectively, which require high computational performance (Stouffer et al., 2007). To calculate all significant network motifs of three nodes within the 48 TS-ENs, we used Mfinder v1.20 (Milo, 2002) with default options.

### Network Statistics

To further evaluate the topological features of the 48 TS-ENs, we developed a software package called NetAn: Network Analyzer^3^ that was built on broadly used python libraries that are freely available, such as Networkx (Hagberg et al., 2008). The main script *NetworkAnalysis.py* receives a list of interactions weighted or unweighted (-d and –u options, respectively) and computes several metrics. We focused on identifying key features that are showed to be significant in comparison with those in random networks. Therefore, we extracted properties of the directed networks such as density, hubs with maximum in-degree and out-degree and clustering coefficient, among others. Then we assumed the networks as non-directed and calculated further topological features such as modularity and communities using the Louvain method (Blondel et al., 2008).

Given that the real networks are very dense in terms of connections, we implemented a method to generate random networks that resemble the real ones using the option gnm_random_graph from Networkx python module. A hundred random networks were generated for each real one, with the same number of nodes and edges. Then, for each one same topological features were extracted and the average compared to those of real networks.

## Results

### Study Field Site Overview

Lagunita pond shows a conductivity ranging from 6.47 to 11.59 mS cm^-1^. At the end of the study, May 2014, salinity was almost double in concentration than in the year before, May 2013. Nevertheless, pH remained constant ≈8 for 2 years. Water physicochemical parameters during wet conditions are shown in Supplementary Table 1, and nutrients for Lagunita were extensively reported by Lee et al. (2015, 2017).

### Metagenomic Analysis

The dataset comprising 12 libraries, consisting of more than 22 million read-pairs, of which ∼9.3% were discarded during quality control. The filtered reads were subsequently assembled, yielding 4,685,929 contigs (N50 ∼417 bp ± 34.36 std). Around 426,300 ± 116,000 std. proteins were detected with Prodigal (see details in Supplementary Table 2), which were then scanned against the Pfam-A v30 database for metabolic inference.

### Microbial Mats Diversity and Community Structure

**FIGURE 2:**
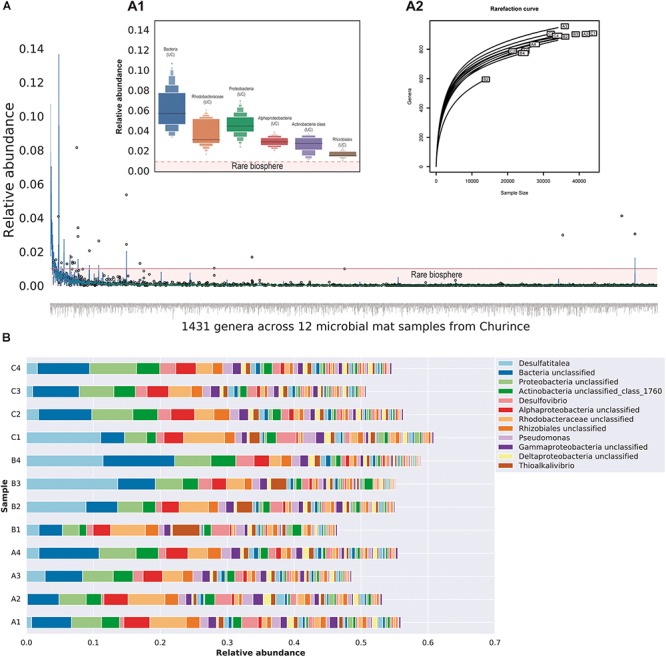
Taxonomic diversity within microbial mats from Lagunita pond at genera level. **(A)** Boxplot distribution showing the relative abundance of the total genera found within microbial mats from Churince where each boxplot represents the distribution of each genera across 12 samples. The horizontal red line indicates the low abundant taxa or RB <0.01 of relative abundance. The inner figure **(A1)** indicates the distribution of the six most abundant genera (>0.01 relative abundance). The second inner figure **(A2)** shows the rarefaction curves of the 12 microbial mats at genera level. **(B)** Stacked bar plots of 58 members of the microbial mat core whose abundance is >0.001 for comparative purposes, this plot shows how the actual composition of each site **(A–C)** change over time (four time points).

Despite the relatively low MiSeq coverage for the twelve metagenomes, we were able to identify 100 bacterial phyla, 168 classes, 302 orders, 539 families and 1431 genera. The number of phyla detected exceeds around three times the number of taxa observed in equivalent studies within mats from Lake Clifton, Australia (Warden et al., 2016), indicating highly diverse microbial mats occur in CCB. Within the total genera identified, 97.27% are found in low abundance ≤0.01 consistently across samples (Figure 2A), and the unclassified sequences are from the Bacteria domain, Rhodobacteraceae family, Proteobacteria phylum, Alphaproteobacteria, and Actinobacteria classes as well as Rhizobiales order, where the abundant taxa among samples with a relative abundance ≥0.01 (Figure 2A1). According to the rarefaction curves, the taxonomic assignment at genera level tends to reach an asymptotic behavior for almost all samples, with the exception of B1 since this sample had a lower number of raw reads (See Supplementary Table 2).

From the total genera identified, 373 were present across all samples, regardless of geographical distance and contrasting environmental conditions (Supplementary Table 3). This subset of taxa could be potentially associated with the microbial mat core. For visual comparisons, the overall abundance shift of the members of the microbial mat core across time are shown in Figure 2B. The vast majority of the core members (344 genera) belong to the RB indicating the presence of permanent rare taxa within microbial mats. Despite their lower abundance (<0.01), RB taxa show a constant presence across samples in all sites during the 2-year study period; hence they cannot be associated with sequencing errors or under-sampling. As expected from previous studies at CCB, a considerable proportion of the members of the microbial mat core, 30% (114 genera), belongs to unclassified sequences. It is worth mentioning that deeper sequencing could reveal a larger core than presented here, for example taxa at lower abundance in all samples.

### Ecological Diversity Index

The alpha-diversity estimators across sites are shown in Supplementary Figure 1. In general, we observe that Site B is the less diverse, especially during dry conditions. Unexpectedly, microbial mats from site A are more diverse at the taxonomic level. The observed differences in richness and diversity between Chao and Shannon indexes may be due to the Shannon algorithm falling short when examining a large number of low abundant organisms (the RB), that in our case represent most of the taxa in the microbial mats. The trajectories of Shannon and Pielou indexes during the period of study indicate little variations of the three sites, pointing out a resilient microbial mat community (Supplementary Figure 2).

**FIGURE 3:**
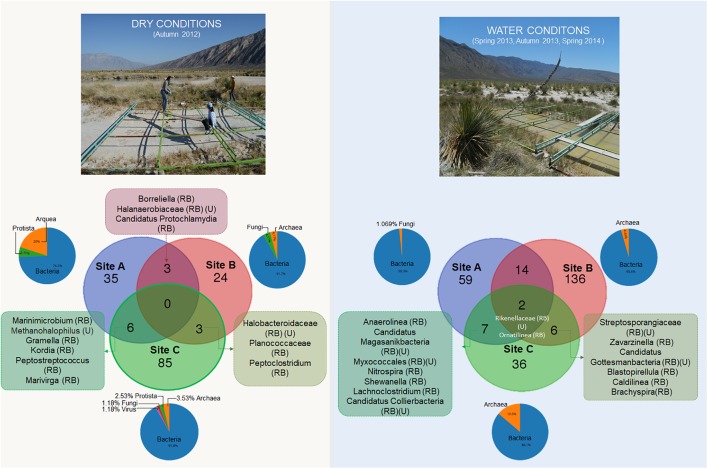
Venn diagram analysis showing the shared and unique taxa at genus level during contrasting water conditions (RB) rare biosphere, (U) unclassified.

Due to the high percentage of low-abundance taxa, we used the genera presence/absence profile to highlight unique and shared taxa during contrasting water conditions (Figure 3).

During dry conditions we detected a diverse community of bacteria, but also Archaea, fungi, protists and lesser number of viruses. Furthermore, we observed the common presence of halophilic taxa, fungi, and protozoa during dry conditions in the three sampling sites (a detailed list is included in Supplementary Table 4). Interestingly, during dry conditions the shared taxa are mostly from the RB, with the exception of *Methanohalophilus* which was common between mats from sites A and C. These two sites in dry conditions also shared six taxa that were never detected during wet conditions. These taxa highlight the implication of a halotolerant and C-cycling community in these mats (i.e., *Gramella* and *Kordia* genus) (more details in Supplementary Table 4).

**FIGURE 4:**
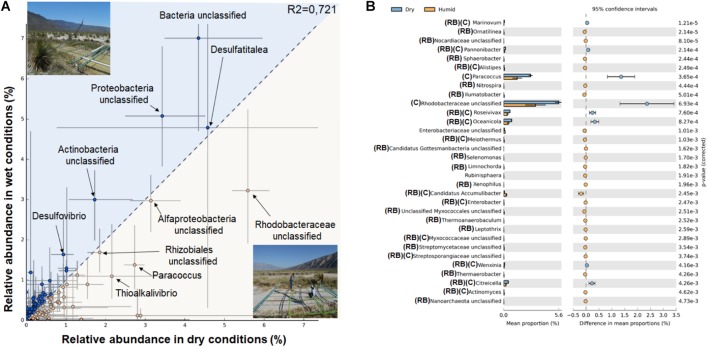
Taxonomic statistical differences between dry and wet conditions. **(A)** Profile scatter plot of each site (A–C), x-axis (dry conditions) and y-axis (wet conditions) microbial mats with the difference in mean proportion among microbial mats within each site along with the associated confidence interval of this effect size (2th and 98th percentile). Points on each side of the gray dashed y = x line are enriched in one of the two samples, SD for proportion are shown as horizontal lines. A statistical hypothesis test is required to determine if the observed difference is large enough, to discount it being a sampling artifact safely, however, in dry conditions there is only one mat for each site therefore no *p*-values are indicated. **(B)** The error bar indicating all genera where Welch’s *t*-test with confidence interval method DP Welch’ inverted of 0.95 produces a *p*-value (<0.005). The difference in mean proportion between the microbial mats during dry and wet conditions are shown in blue and orange, respectively (RB, rare biosphere; C, microbial mat core).

During wet conditions, representatives of fungi and protista were not found within wet patches, contrasting with site dry mat, where we observed a genus of the Leotiomyceta class (Ascomycota) (details in Supplementary Table 5). Interestingly, given the small spatial scale, many unique taxa exist in each separated community (especially in site B). In contrast to dry conditions, we found two taxa that are common in all the sites in the water conditions (*Ornatilinea* and Rikenellaceae) they belong to the RB (Figure 4).

### Statistical Analysis

The analyses made to identify the proportion of sequences assigned to each genus that was enriched among dry and wet conditions (first versus other sampling points Figure 4) indicate that during dry conditions, unclassified sequences from Rhodobacteraceae, Alphaproteobacteria, Rhizobiales, *Paracoccus*, and *Thioalkalivibrio* were consistently over-represented in the three site points. However, when water conditions were restored unclassified sequences from Bacterial domain, Proteobacteria and Actinobacteria, as well as sulfate reducers (*Desulfovibrio* and *Desulfatitalea*), were enriched (Figure 4A).

To gain an insight into the low abundant taxa enriched during dry and wet conditions in Figure 4B we show the extender error bar in Welch’s *t*-test to highlight the differentially abundant (*p* < 0.005 genera). In the case of dry conditions eight genera were observed: *Marinovum, Pannonibacter, Paracoccus*, unclassified Rhodobacteraceae, *Roseivivax, Oceanicola*, *Wenxinia, and Citreicella*. In contrast, we observed a total of 24 genera enriched during wet conditions, including the potential phosphorous removal by Candidatus Accumulibacter, unclassified sequences from Nanoarchaeota, and *Ornatiline*a.

Specific statistical taxonomic differences across sites suggests a clear involvement of anaerobic taxa in mats from wet patches, whereas in the dry site we observed mostly photosynthetic and heterotrophic genera. Among the differentially abundant genera from microbial mats from site C compared with those from site A we found several representatives of sulfate reducing bacteria such as *Desulfococcus, Desulfosarcina, Desulfonatronospira, Desulfatibacillum, and Geoalkalibacter*. In contrast, site A was enriched with marine-related genera such as *Plesiocystis, Hyphomonas, Enhygromyxa, and Arenimonas* (Supplementary Figure 3). The same pattern was observed in mean proportions between mats from site A and B, being enriched with the genera *Spirochaeta*, unclassified candidatus *Daviesbacteria, Desulfonatrospira, Desulfobacterium* in site B, whereas unclassified cyanobacteria along with *Synechococcus, Nostococales*, and *Oscillatorial*es are differentially abundant within site A (Supplementary Figure 4).

When comparing the differentially abundant genera from wet patches (site B and C), we observed a representation of an aerobic community enriched within site C (*Rhizobacter, Rhodospirillum, and Skermanella*) (Supplementary Figure 5). Despite the geographic closeness of the three sites, each one presents many elements unique from each community and a unique response of such taxa for perturbation being sulfate reducers common in wet patches.

**FIGURE 5:**
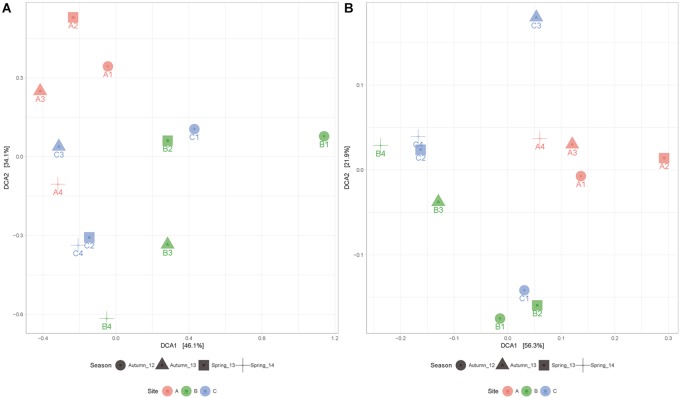
Detrented correspondence analysis (DCA) according to taxonomic **(A)** and metabolic **(B)** composition of microbial mats explaining 80.2 and 78.2% of the variance among samples, respectively. These results are supported by PERMANOVA analysis.

PERMANOVA analysis indicates that the microbial mats have statistically different taxonomic composition (*p* < 0.05) depending on site and season, despite having several enriched taxa in common during contrasting water conditions. Moreover, when we compare samples metabolic composition, we found significant differences between sites (*p* = 0.005) but not across seasons (*p* = 0.072), indicating that in every site, the metabolic potential was maintained over time. This pattern can also be observed in detrending correspondence analysis (DCA) (Figure 5) where distribution of samples according to metabolic functions are clustered principally by site.

### Dynamics of the Main Cycles Over Time

**FIGURE 6:**
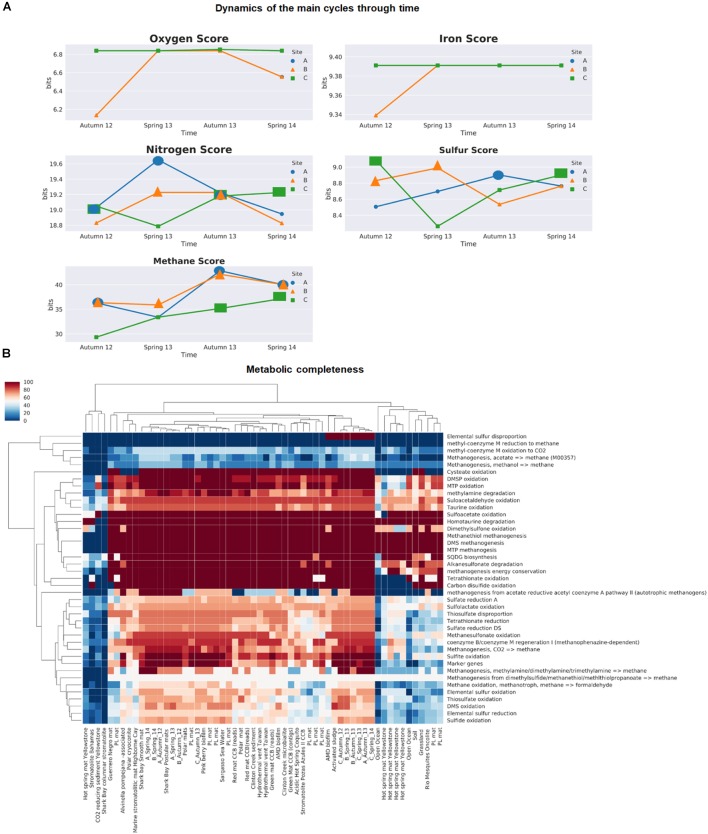
Biogeochemical cycling within microbial mats across space and time using MEBS. **(A)** Dynamics of the main cycles within microbial mats samples during the two-year period of study by with a single value MEBS captured in bits. **(B)** Metabolic completeness in a color gradient, the more complete are red and the less shift to blue. Sulfur and methane cycle across 58 environments including those analyzed in this study and several environments such as hydrothermal vents, biofilms, microbial mats, stromatolites and soils. Samples from this study are named according to the site (A–C), and sampling season and year.

For comparative purposes, we used a restrictive FDR to avoid false positives and to detect the most enriched cycles within the microbial mats. By using this approach, we observed that methane and nitrogen cycles were over-represented in the microbial mats (with asterisks in Supplementary Table 6 and bigger markers in Figure 6).

In the first case, the methane cycle, characterized by the usage of CH_4_ compounds by methanotrophs, methanogens, and methylotroph, is always enriched within site B and becomes significant in all microbial mats after the aquifer recovery at the end of the period of study.

The dynamics of the nitrogen cycle, which includes pathways involved in the reduction and oxidation of both inorganic [nitrate (+5) to ammonia (-3)] and organic nitrogen compounds (i.e., taurine, urea, and choline degradation), follows an opposite behavior, since it is significant in all samples at the third sampling time in October 2013 (Supplementary Table 6 and bigger markers in Figure 6). This is interesting since that sampling period corresponds to a moment of recovery of the aquifer after the closing of a canal that was draining the site. Furthermore, site C is the only one that becomes significant at the end of the period of study compared to the rest of the mats.

In our previous benchmark for the sulfur cycle (Figure 6 in De Anda et al., 2017), we observed that microbial mats from Churince CCB were among the most enriched in terms of sulfur cycle compared with nearly 1000 metagenomes, with a sulfur score above the 95th distribution (>8.81 bits) are shown with bigger markers in Figure 6A. We observed a tendency that indicates that during dry conditions in site A, the overall machinery of the sulfur cycle was underrepresented compared to the wet patches sites B and C. These results agree with the anaerobic differentially abundant taxa within wet patches, suggesting that the water fluctuations largely determine the redox gradient within the mats affecting the anaerobic organisms.

In the case of the oxygen and iron cycle (Figure 6A), our results indicate that there is neither enrichment nor impoverishment of the above-mentioned cycles in the microbial mats from Lagunita pond. For example, in the case of photosynthesis (oxygen score), our results agree with the low proportion of cyanobacteria detected in the taxonomic profile, indicating an under representation of the oxygenic photosynthetic pathways in our community during the period of study. The iron cycle, represented by the reduction and oxidation of iron compounds including siderophores uptake, was under-represented during dry conditions within site B, but then become stable over time.

In general terms, the comparison of MEBS scores indicates that the sulfur, methane, and nitrogen cycles are over-represented in microbial mats from Lagunita, as well as in Pavillon Lake (White et al., 2016), both sites displaying similar patterns. Interestingly, Pavillon Lake is another analog of early Earth studied by NASA, in British Columbia and, as Lagunita, it is a non-extreme continental site with microbialites. However, the over-representation of the methane cycle is evident within microbial mats from our study, compared with the rest of environments including soil, microbialites, hydrothermal vents, biofilms, and open ocean samples (Supplementary Table 6).

For a closer look into the dynamics of sulfur and methane cycles, we employed the completeness module implemented in MEBS. As established previously, the metabolic completeness is defined as the full repertoire of protein domains involved in a given metabolic pathway such as sulfate reduction or methanogenesis (De Anda et al., 2017). As observed in Figure 6B, the complete repertoire of protein domains required to utilize the organic sulfur compounds such as sulfonates as Dimethylsulfoniopropionate (DMSP), [3-(methylsulfanyl) propanoate (MTP)] (Jonkers et al., 1998; Curson et al., 2008; Bullock et al., 2014), as well as the methanogenesis from organic sulfur compounds (methanethiol and DMS) were found in the majority of microbial mat samples with exception of hot-spring mats from Yellowstone and stromatolite from Bahamas. In addition, the dimethyl sulfide (DMS) oxidation pathway reported for aerobic microorganisms (such as *Thiobacillus*, *Hyphomicrobium*, methanogens and sulfate reducing bacteria) was also found to be 100% complete after the disturbance event in microbial mats from Lagunita and in site C during dry conditions.

The elemental sulfur disproportion represented by the sulfur oxygenase/reductase (SOR) protein isolated from the thermoacidophilic and chemolithoautotrophic crenarchaeote *Acidianus ambivalens* (Kletzin, 2008) was only found in microbial mats from Lagunita (after the desiccation event and in a wet patch from site C), and one biofilm from an Acid Drainage Mine (ADM), but was found to be absent in the rest of the metagenomes. The main pathways for oxidation and reduction of inorganic sulfur compounds (sulfate reduction, sulfide, and sulfite oxidation), are 80–100% present in all samples from microbial mats of Lagunita, but their completeness is not always observed in the rest of microbial mats (i.e., Yellowstone, or stromatolites from Bahamas) (Figure 6B).

Finally, we can argue that the fact that the methane cycle is over-represented in our samples, may be due to the majority of main pathways involved in the methanogenesis, as well as the oxidation of methane and methanesulfonate are almost complete in the Lagunita mats, contrary to their poor representation in the rest of the microbialites.

### Network Inference Method Based on Time-Series Data

First, we evaluated whether the regenerated abundance profiles obtained from MetaMIS successfully reproduced the microbial abundance similar to the original by using the Bray Curtis Dissimilarity (BCD). As observed in Supplementary Table 7, a small BCD (mean 0.136 ± 0.021 std.) was obtained in the three sites at all taxonomic levels, suggesting that interactions revealed successfully the underlying interactive relations of the microbial mat communities. We also confirmed that the majority of taxa within microbial mats were found to be rare <0.1% (75% ± 3.06 std.).

From 1600 intermediate networks generated by MetaMIS, we obtained 48 TS-ENs that comprised the total number of interactions at every taxonomic level (Supplementary Table 7 for details). We used NetAn to compute the several topological properties of real and random networks. Due to the high number of relationships (highly dense networks) and the method used to generate the random networks, it is not surprising that the structure of both real consensus and random networks are similar (Table 1). This data suggests that a large number of taxa (probably from the diverse RB) co-occur randomly, with exception of those that belong to the microbial mat core. This also suggests that this large RB co-occurs randomly, probably being part of the large and dynamic seed bank of the deep aquifer. Although several methods were tested to generate the random networks (data not shown), the values were similar to the real ones, indicating that small world properties (Zhou et al., 2010) do not characterize our TS-ENs.

**Table 1:** Global network measures obtained from real (consensus) and random networks Std. of the values obtained across the span of taxonomic levels from Phylum to Family.

	Site A		Site B		Site C	
	Real	Random	Real	Random	Real	Random
Clustering Coefficient	0.9620 (±0.0247)	0.9268 (±0.0497)	0.9507 (±0.0034)	0.9106 (±0.0090)	0.9597 (±0.0069)	0.9288 (±0.0278)


Density	0.927 (±0.043)	0.9269 (±0.0498)	0.911 (±0.008)	0.9106 (±0.0090)	0.892 (±0.024)	0.8920 (±0.0278)
Diameter	2.000 (±0.707)	1.9625 (±0.0750)	2.000 (±0.000)	2.000 (±0.000)	2.500 (±0.500)	2.000 (±0.000)
Modularity	0.003 (±0.002)	0.0010 (±0.0014)	0.002 (±0.002)	0.0005 (±0.0008)	0.004 (±0.002)	0.0015 (±0.0014)
Radius	1.250 (±0.433)	1.1250 (±0.2500)	1.250 (±0.433)	1.0000 (±0.0000)	1.500 (±0.500)	1.0625 (±0.1250)
Mean degree	159.391 (±97.214)	159.3912 (±112.2527)	164.246 (±88.870)	164.2461 (±102.6182)	168.933 (±92.229)	168.9333 (±106.4964)
Communities	2.500 (±1.500)	1.7475 0.9531	2.250 (±1.090)	1.5600 (±0.5931)	3.000 (±1.581)	2.0850 (±0.8296)

Our results indicate that microbial mats are more robust to perturbation due to redundant functions that are likely shared among nodes or functional clades in the highly connected TS-ENs, with density values close to one (≈0.9) (Montoya et al., 2006; Steele et al., 2011; Faust and Raes, 2012a; Shaw et al., 2016). Consensus and negative networks displayed a higher clustering coefficient (∼0.9 and ∼0.7 respectively), while positive interaction networks showed much lower values (≈0.2–0.3). Networks with a high clustering coefficient are likely to contain more hubs or focal nodes than those with a lower coefficient (Proulx et al., 2005; Peura et al., 2015). Hence, the loss of these hub nodes, which have been likened to ‘keystone’ species (Steele et al., 2011), reflects potential structural perturbations to the community and suggests some degree of community stress as bacterial associations have been fractured.

Our data indicates that negative relationships within mats may retain a greater number of hubs than positive interactions, suggesting an important role for competitive interactions in stressed conditions. As indicated by Grilli et al. (2016), positive modularity values indicate that interactions occur predominantly within groups, while negative values indicate that interactions are more frequent among groups. In our study, the consensus networks have associated positive values of modularity close to zero, indicating that taxa within microbial mats prefer those members from another network subsystem due to the highly densely connected network. However, when we separated the consensus networks by their type of interaction (+ or -), we found a subtle modularity increase to 0.2 in the negative networks, suggesting a preference for competitive interactions within the same subsystem (Table 2).

**Table 2:** Global network measurements of random and real networks.

	Negative interactions			Positive interactions		
	A	B	C	A	B	C
Clustering coefficient	0.7334 (±0.020)	0.5838 (±0.025)	0.7139 (±0.026)	0.3270 (±0.038)	0.1953 (±0.020)	0.2580 (±0.022)
Random	0.5745 (±0.025)	0.5920 (±0.008)	0.4482 (±0.012)	0.3522 (±0.029)	0.3184 (±0.013)	0.4457 (±0.030)
Modularity^∗^	0.09 (±0.02)	0.13 (±0.02)	0.24 (±0.01)	0.05 (±0.01)	0.06 (±0.02)	0.02 (±0.01)
Random	0.025 (±0.005)	0.023 (±0.005)	0.037 (±0.011)	0.052 (±0.013)	0.057 (±0.017)	0.037 (±0.007)
Diameter^∗^	2.25 (±0.50)	2.50 (±0.58)	2.50 (±0.58)	2.25 (±0.50)	2.50 (±0.58)	2.50 (±0.58)
Random^∗^	2 (±0.0)	2 (±0.0)	2 (±0.0)	2 (±0.0)	2 (±0.0)	2 (±0.0)
Number of hubs with max in degree	3.50 (±2.38)	2.25 (±0.96)	6.50 (±7.33)	1.50 (±1.00)	2.25 (±2.50)	1.25 (±0.50)
Random	1.32 (±0.13)	1.27 (±0.14)	1.29 (±0.13)	1.26 (±0.11)	1.26 (±0.04)	1.26 (±0.11)
Number of hubs with max out degree	1.00 (±0.00)	1.75 (±0.50)	1.00 (±0.00)	1.75 (±1.50)	1.50 (±1.00)	6.50 (±10.34)
Random	1.29 (±0.13)	1.22 (±0.06)	1.23 (±0.12)	1.20 (±0.07)	1.28 (±0.11)	1.21 (±0.09)

*Std (±) represent the values obtained across the span of taxonomic levels from Phylum to Family. Properties marked by ^∗^ are calculated on the underlying non-directed network*.

Our results indicate that modularity in our networks was very low compared to other reported biological networks (Baldassano and Bassett, 2016). The low modularity values indicate that our TS-ENs lack an evident modular architecture. Therefore, we can hypothesize that modularity values close to zero in the studied microbial mats point toward a community behavior as a complete module, where all the members are interacting with each other.

By comparing the properties of the TS-ENs by their type of interaction (positive and negative) against the random ones (Table 2), our data indicates that negative relationships may retain a greater number of hubs than positive interactions, highlighting again the important role of competitive interactions within the mats. We also found that modularity increased in the negative associations, suggesting large groups of mutually excluding taxa and a clear dominance of competitive interaction within the same subsystem, in agreement with the clustering coefficient mentioned for the consensus TS-ENs.

**FIGURE 7:**
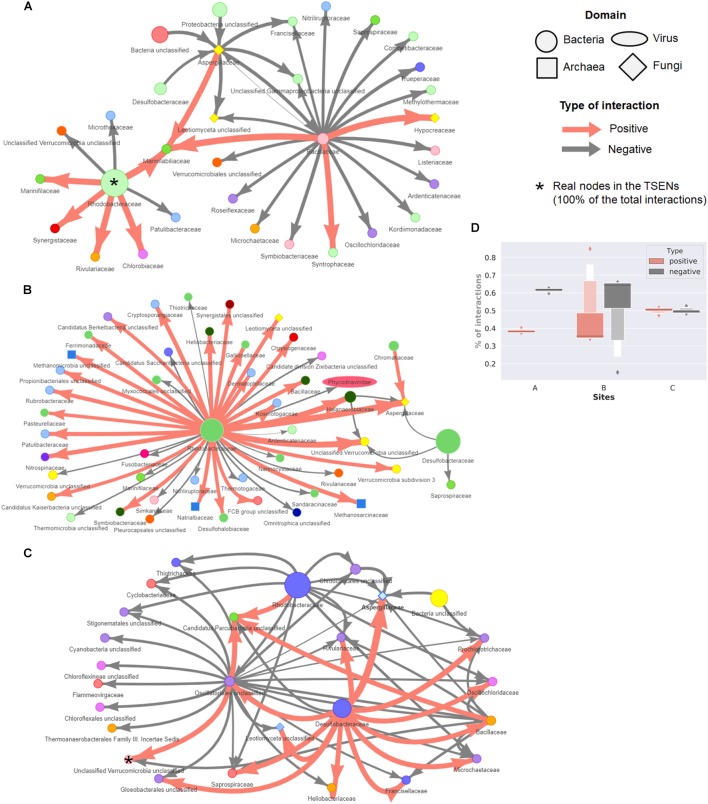
Network representation of the consensus networks displaying only to top 0.05% of total interactions. These interactions represent less than 50 from around 450 consensus families for three sites. In site **(A)** are displaying 61/122,841 interactions, from which 17/46,200 are positive and 44/76,641 negative. In site **(B)** 53/107548, interactions are shown, being 28/37, 170 positive and 25/70,378 negative. From microbial mats of site **(C)**, the consensus network is composed from 60/121,056, from which 16/57,487 are positive and 40/63,569 negatives. The size of a circle (node) is proportional to the abundance of the family across the microbial mats from each site. The thickness of a connection (edge) is proportional to the strength of the interaction. Families are colored according to the Phylum. **(D)** Distribution of the percentage of positive and negative interactions in the consensus TS-ENs of each site.

The consensus TS-ENs at family level are shown in Figure 7. To facilitate visual representation, we displayed only the 0.05% of the top relationships. The latter is done since the networks displaying the 100% of the total interactions are so densely connected that the network patterns and hubs cannot be appreciated clearly. At this level of low-resolution, we observed a crucial role of the family Rhodobacteraceae as a hub within the three sites, having more negative associations within site C. The family Desulfobacteraceae is also a key component within wet patches, having both negative and positive relations with fungi representatives (Aspergillaceae) in site B and C, respectively. However, these hubs came to light only when we focused on a small percentage of relationships. Therefore, in order to identify whether the hubs observed in this low level of resolution, we identified the highly connected nodes either affecting (max out-degree) or being affected (max in-degree) by many links in the consensus, positive, negative, and global TS-ENs. Those hubs found in the consensus TS-ENs and in the low-resolution networks are highlighted with asterisks in Figure 7. In order to infer possible metabolic roles of the potential key stone taxa, we focused only on the nodes at family level, identifying those that were either part of the microbial mat core (C) rare biosphere (RB) or capable of delivering public goods (PG) to the system with prior reported evidence to perform such task (Table 3).

**Table 3:** Strongly interconnected taxa or hubs identify at family levels within TS-ENs of microbial mats.

Site	Type of interaction	Max in-degree (affected by *n* nodes)	*n*	Max out degree (affecting *n* nodes)	*n*
**A**	*Consensus*	1. Nannocystaceae (PG)(RB)	337	1. Polyangiaceae (PG)(RB)	345
		2. Desulfarculaceae (C) (PG) (RB): *Desulfarculus*			
		3. Bacteroidaceae (C)(RB): *Bacteroides*			
		4. Caulobacteraceae (C)(RB): *unclassified Caulobacteraceae*			
		5. Bradyrhizobiaceae (C): Bosea, unclassified Bradyrhizobiaceae, Bradyrhizobium			
		6. Unclassified Oscillatoriophycideae (UC) (PG)(RB)			
		7. Rhodobacterales (UC)(PG)(RB) 8. Actinobacteria phylum 201174 (UC)(PG)(RB)			

	*Positives*	1. Unclassified Chloroflexi (RB)	275	1. Unclassified Armatimonadetes (UC)(PG)(RB)	263

	*Negatives*	1. Unclassified Rhodobacterales (UC)(RB)	278	1. Pelobacteraceae (C)(RB)(PB): *Pelobacter*	275

**B**	*Consensus*	1. Unclassified Xanthomonadales (UC)(RB)(PG)	318	1. Thermoactinomycetaceae (C)(RB)(PG): Thermoactinomyces	325
		2. Beijerinckiaceae		2. Actinomycetaceae (C)(RB)(PG): Actinomyces	
		3. Burkholderiaceae (C): *Burkholderia, unclassified Burkholderiaceae, Burkholderiales Genera incertae sedis unclassified, unclassified Burkholderiales*		3. Alcaligenaceae (C) (RB): *unclassified* Alcaligenaceae	
		4. Phyllobacteriaceae (C)(PG): *unclassified Phyllobacteriaceae*		4. Gemmantimonadaceae	
		5. Gemmantimonadaceae			

	*Positives*	1. Staphylococcaceae (RB)	226	1. Fusobacteriaceae (RB)	246

	*Negatives*	1. Phyllobacteriaceae (C): *unclassified Phyllobacteriaceae*	298	1. Cystobacterineae (UC)(RB)	289
				2. Cloacimonetes (UC)(RB)	

**C**	*Consensus*	1. Unclassified Verrucomicrobia (UC)(RB) 2. Methylobacteriaceae (C) (PG):*(unclassified Methylobacteriaceae, Methylobacterium)*	335	1. Chromobacteriaceae (C)(RB)(PG): *unclassified Chromobacteriaceae*	343
				2. Mycobacteriaceae (C)(RB):*Mycobacterium*	
				3. Symbiobacteriaceae (RB)	

	*Positives*	1. Unclassified Archaea (RB)	252	1. Micrococcales (UC)(RB)(PG)	223

	*Negatives*	1. Rhizobiales (UC)	251	1. Bacteroidetes (UC)(RB)	222
		2. Alteromonadaceae			
		3. Methylococcaceae (C)(RB): *unclassified Methylococcaceae*			
		4. Bacillales (UC)(RB)			
		5. Corynebacteriales (UC)(RB) 6. Beijerinckiaceae (RB)			

**Global (A+B+C)**	*Consensus*	1. Desulfarculaceae (C) (RB): *Desulfarculus*	391	1. Bacteroidales (UC)(RB)	393
		2. Pelobacteraceae (C)(RB): *Pelobacter*		2. Microgenomates group (UC) (RB)	
		3. Gemmatimonadaceae		3. Nocardiaceae (C) (RB): *Nocardia*	
				4. Nitrospiraceae (RB)	
				5. Prolixibacteraceae (RB)	

	*Positive*	1. Moraxellaceae (RB)	349	1. Unclassified Eukaryota (UC)	352

	*Negatives*	1. Phyllobacteriaceae (C): unclassified Phyllobacteriaceae	367	1. Desulfobacteraceae (C): *Desulfatibacillum*, *Desulfatiglans*, *Desulfatirhabdium*, *Desulfatitalea, Desulfobacter*, unclassified Desulfobacteraceae, *unclassified* Desulfobacterales	362

*C, core microbial mat (presence in all samples regardless of environmental conditions and abundances). UC, unclassified sequences belonging to the microbial mat core. PG, taxa with known members delivering public goods (i.e., metabolites, enzymes, and vitamins). RB, rare biosphere <1% (0.01) abundances*.

In the case of site A, we identified in the consensus TS-ENs eight hubs affected by 337 nodes, indicating a high interconnectivity within the overall system, and one hub affecting 345 nodes represented by the Polyangiaceae, a type of myxobacteria that is known for excreting hydrolytic enzymes and decomposing various and complex biopolymers (Brinkhoff et al., 2012). Interestingly, the number of hubs in the positive and negative networks decreases to some keystone taxa that belong to both the RB and the microbial mat core (see details in Table 3).

Our results indicate that the number of keystone taxa affected by, or affecting other members in the microbial mat community within site B is equivalent, being the majority members of both RB and microbial mat core. An interesting example within these hubs is the family Thermoactinomycetaceae, which is known for its protein degradative capacities, strong lipolytic activity, and alpha-amylase activity, as well as antimicrobial activity (Frikha Dammak et al., 2017).

By analyzing the consensus network from the microbial mats of site C, we found two hubs belonging to the microbial mat core; one is Verrucomicrobia, a lineage that is also part of the RB. Interestingly, this phylum is known to perform saccharolytic lifestyle commensal and mutualistic relationships with ciliates (Vannini et al., 2003).

Unexpectedly, within the positive networks, we observed a hub of unclassified sequences from the Eukarya domain whose presence could be potentially associated with an increase in the energy transfer, and therefore trophic complexity and potential resilience to environmental change (Duffy and Stachowicz, 2006).

For a visual comparison between low-level resolution networks and the consensus TS-ENs, we focused on the total amount of positive and negative interactions within sites. As observed in Figure 7D, site A displayed a larger percentage of negative interactions (0.616 mean ± 0.012 std.) compared with the positive ones (0.384 ± 0.011). In contrast, in the wet patches (especially in site C) approximately half of the interactions were found to be equally positive and negative (considering only average values).

### Network Motifs

**FIGURE 8:**
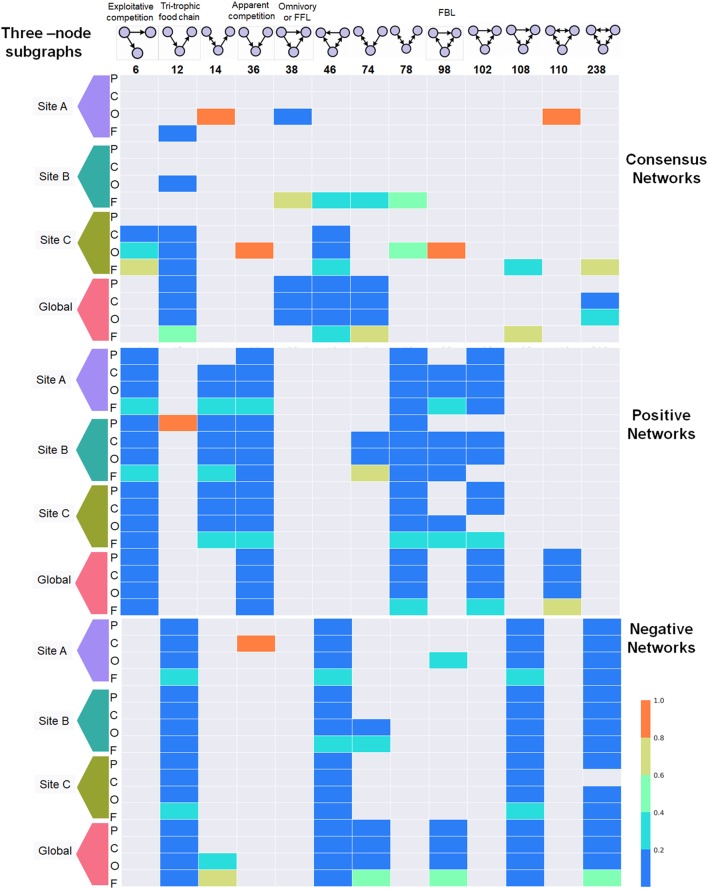
Distribution of the 3-three-node subgraphs or network motifs across 48 microbial mat networks built across the taxonomic levels: Phylum (P), Class (C), Order (O), and Family (F). The top panel represents the motifs sorted by their ID identifier described in Mfinder. The motifs with specific ecological terminology are ID6, ID12, ID36, and ID38. The latter motif is also known as Feed Forward Loop (FFL) in regulatory networks. The ID98 represents Feedback Loop (FBL). To facilitate visual comparison, the abundance of each motif was normalized by its appearance across taxonomic levels. The relative abundance of each one indicates that a given motif is only found in that particular taxonomic lave (i.e., id 36 Site A in negative networks at class level). It is observed that particular motifs appear over-represented across the span of taxonomic levels when consensus networks are separated by type of interaction either positive or negative (i.e., Motif 108 in negative networks or motif 36 in positive ones). The color code in the bars indicate the scale from over representation (red) of a given motif in a given taxonomic level shifting to underrepresented (blue).

We focused on the representation of three-node motifs over 48 TS-ENs to observe whether these motifs could reflect the behavior of microbial mats from three sites during and after water depletion. In our study, we considered only those motifs whose probability of appearing is lower than a cutoff value (here *p* = 0 and *p* ≤ 0.05) according to the distribution observed in randomized networks (Jin et al., 2007). As observed in Figure 8, these motifs appear numerous times on each particular network at every taxonomic level. For visual comparison, we normalized the abundance of each motif by the sum of the total across taxonomic levels. Therefore, an abundance of one indicates that a given motif is only found in that particular taxonomic level.

The occurrence of each motif can be observed consistently across the span of taxonomic levels when consensus networks are separated by type of interaction, either positive or negative. For example, motifs 6, 36, 78, 102, and 110 are under-represented in the negative TS-Ens, but are highly distributed across the positive interactions. This is particularly interesting since motif 6 (exploitative competition) occurs when some quantity of resources is consumed by an individual, thereby depriving other individuals of it. This type of competition has also been called consumptive competition (Kawata, 1997), and was never found among the negative TS-ENs within our microbial mats. The latter indicates that motif (6) can potentially have a different ecological connotation in microbial ecology, for example one bacteria delivering PG to the system. The same pattern is observed for the apparent competition motif (36) that occurs when two individuals that do not compete directly for resources affect each other indirectly as prey for the same predator (Lang and Benbow, 2013). However, in a microbial ecological context, the fact that this type of motif is mainly distributed across positive relationship could indicate beneficial relationships from two partners. For example, in the case of microbial mats from CCB, we suggest that this motif could correspond to a sulfate reducing bacteria using both organic compounds from a cyanobacteria and sulfate derived from purple bacteria to be used as the terminal electron acceptor in an energy-gaining respiratory process.

Furthermore, we found that motifs 12, 46, 108, and 238 are widely distributed across negative TS-ENs, but under-represented within the positive ones. These motifs seem to display more complex relationships, for example motif 238 represents the most mutually exclusive relationships in which all nodes exclude each other in a cycle form. Consistent with the ecological theory (Stouffer et al., 2007), the tri-trophic food chain motif (composed by prey, predator, and top-predator) was over-represented within negative networks compared to positive ones. However, since we also found the same motif in the positive interactions and in the consensus networks, it is possible that in our communities it represents both beneficial and negative interactions (Baiser et al., 2016).

The FeedForward-Loop (FFL) motif is one of the most significant motifs in *E. coli* and yeast, representing a structural stable motif with no feedback interaction and it has been found to be over-represented in transcriptional networks (Shen-Orr et al., 2002; Mangan and Alon, 2003). In ecological theory, the FFL involves omnivory, representing a predator consuming species from two lower trophic levels (Stouffer et al., 2007; Bascompte and Stouffer, 2009). In our TS-ENs, this motif must have at least one positive and one negative interaction since it is only found in the consensus TS-ENs. Another example is the Feedback-Loop (FBL), which has been recognized as an unstable network motif that is under-represented in gene regulatory neuronal networks (de-Leon and Davidson, 2007). However, in our study, we observed an overrepresentation of this motif within the positive networks that could be an example of positive metabolic coupling occurring within microbial mats.

Finally, under the hypothesis that the environment can influence the strength and type of interactions between taxa, we expected to find similar interaction patterns in the microbial mats isolated from the wet patches. Interestingly, we only found that site B displayed a specific type of relationship, -the network motif ID74-. The latter indicates that specific local roles within site B that are not found within the microbial mats from the other sites, highlighting a very specific community dynamic and structure, even in the presence of the same environmental constraints.

## Discussion

The effects of wet versus dry conditions in Lagunita pond in Churince, CCB, on microbial communities were the main focus of our study. We observed that the lack of water in one site disrupted the fundamental characteristics of well-developed microbial mats, where the redox gradient includes a deep anaerobic environment that is fundamental for its function and stratification (Sorokin et al., 2006; Grimm et al., 2011; Higashioka et al., 2013; Eren et al., 2014). In contrast to normal wetland conditions, microbial mats from site A (the dry patch) were exposed to bright sunlight, in direct contact with the atmosphere losing their redox gradient and leading to a harsh photo-oxidative microenvironment at their surface. Given the remarkable physicochemical-zonation observed in microbial mats characterized by steep vertical gradients of oxygen, pH and sulfide (Chennu et al., 2015). Considering that metabolite exchanges in microbial communities give rise to ecological interactions that govern ecosystem diversity and stability (Zomorrodi and Segrè, 2017). It is expected that the lack of water has affected not only the community composition, structure and function, but also the community-level relationships.

Comparing our data with the analysis of microbialites from Pavilion lake (White et al., 2016), we found that they are supported by carbonate-rich structures associated with bacteria producing exo-polysaccharides (EPS). Here cyanobacteria are important in the organo-mineralization via dissimilatory sulfate reduction that precipitates compounds in carbonate stromatolites (White et al., 2016). Meanwhile, in our study system there is mostly gypsum and the mats at Lagunita are soft. Nevertheless, it is possible that a type of organo-mineralization occurs in wet conditions since we found cyanobacteria as well as unclassified sequences from Bacterial domain, Proteobacteria, Actinobacteria, and sulfate reducers such as *Desulfovibrio* and *Desulfatitalea* (also conforming the core and RB). This is interesting because in Pavilion Lake, the Deltaproteobacteria were assigned to the same genera of dissimilatory sulfate reducers that we observe in Lagunita (e.g., *Desulfobacterium* and *Desulfovibrio*). This suggests that even though the shape and consistency of the microbial communities are different, they are functionally similar to other marine and freshwater mats despite geographical and environmental differences.

### Freshwaters Microbial Mats From Lagunita Are Highly Diverse

The large number of identified taxa (100 Phyla), mainly represented by low-abundant members within microbial mats indicate that this environment has a rich “seed pool” of genetic diversity. The latter suggests not only a large biological complexity at the micron scale (Minz et al., 1999a,b), but also a very dynamic structure with different ecotypes with apparently overlapping ecological features. As expected by the ecological theory on perturbations (Eng and Borenstein, 2018), the modifications of environmental conditions lead to changes in the proportion of species members in the community (measured at genera level).

When we explored the relationship between taxonomic and metabolic diversity estimated with ecological metrics (i.e., Shannon, Pielou, rarefaction curves) and the functional potential for several biogeochemical cycles by using MEBS algorithm, site A (dry in November 2012) was more diverse and resilient over time than expected, maybe because it was a fluctuating environment with wet winters until 2014. However, we believe that such resilience has been lost now that the lagoon has been permanently dry since 2016. Interestingly, even if wet patches do not display the same taxonomical dynamics as dry patches, they retained functional similarity. This functional core may be fulfilled by different key members of the RB. Another possible explanation for these results is the micro-dynamics of the deep aquifer. It is possible that site A was a little bit further from the micro-filtrations of deep water then the two neighboring sampling points (B and C), making it drier as the aquifer became depleted. Nevertheless, fluctuating environments have been shown to promote diversity in the different studied CCB environments (Bonilla-Rosso et al., 2012; Peimbert et al., 2012; Pajares et al., 2015).

### Metabolic Dynamics in the Freshwaters Microbial Mats From Lagunita

By using MEBS, we were able to capture the fluctuation dynamics of the whole metabolic machinery involved in the main cycles, not only by focusing on a few marker genes, but rather by following the importance of the overall behavior over time. Our results indicate that the anaerobic cycles within microbial mats from the wet patches are maintained by the redox conditions probably related with the deep sulfur rich aquifer. This becomes more apparent when the aquifer recovered in 2013, due to the temporal closing of a canal 5 km away. When the water returned, the sulfur cycle became over-represented within the dry patch (site A, Autumn 2013). This is also evident in the potential methane cycling, where we observed microbial mats from the three sites were over-represented during the last two sampling periods (Autumn 2013 and Spring 2014) when water returned to the Lagoon.

Despite having no metagenomic samples before Autumn 2012, the Churince within CCB has been our focal study site for more than 15 years (Cerritos et al., 2011; Lee et al., 2015, 2017; Ponce-Soto et al., 2015). Long-term observations have seen the slow but dramatic decay of the ecosystem from a well-defined wetland to a nearly a dry desert with only a small spring and sections of the former river (Souza et al., 2007, 2018; De Anda et al., 2018). Sadly, this study was the last chance to understand the effect of perturbation and recovery on microbial mats from Churince, unless changes water usage policy start to allow the wetland to recover its deep aquifer.

Our results indicate that the sulfur cycle was only significantly overrepresented in microbial mats from site A after water recovery. We can suggest that perhaps site A could have had the potential for sulfur cycling under initial conditions, where low-abundant members of the microbial community were “waiting” the proper ecological conditions to develop particular metabolic features (i.e., elemental sulfur disproportionation in Figure 6B). Another hypothesis is that deep-water microbial migrants filled the empty ecological niche performing key metabolic processes within the mats. However, due to the large diversity of low-abundant members within the mats, we can argue that the “seed bank” hypothesis is more likely to occur, given the representation of the RB as key stone taxa in the TS-ENs.

There are other important keystone taxa that are dominant within mats, especially within dry conditions. For example, we can associate the abundance of the physiologically and metabolically versatile Rhodobacterales to their potential as primordial colonizers for the formation of biofilms in aquatic environments. This can explain their adaptation to dry environments by forming biofilms that are able to resist drying (Dang et al., 2008; Elifantz et al., 2013). Consistent with the visual structure of wet patches, the genera enriched among dry conditions were those implicated in the oxidation of inorganic reduced sulfur compounds (i.e., *Thioalkalivibrio*) which is a physiologically and metabolically taxon adapted to hyper-saline (up to saturation) and alkaline (pH up to 10.5) conditions (Foti et al., 2006; Sorokin et al., 2011). In contrast when the water levels were recovered, we detected an unknown diversity of unclassified Proteobacteria, along with the sulfate reducing bacteria *Desulfatitalea* [that is part of the microbial mat core and common in marine sediments (Higashioka et al., 2013)]. Our results confirm previous findings showing that sediments of the hypersaline lakes and lagoons may support a rich community of anaerobic halophilic bacteria, as the solubility of oxygen in hyper-saline brines is low and the amounts of organic matter available are often high (Oren, 1988, 2008).

Even if there are taxonomic shifts under environmental perturbation in the microbial mats of Lagunita, their resilience and resistance is evident when we compare their metabolic diversity, as the microbial mats originally sampled from wet-patches are metabolically similar. In addition, the anaerobic community that was shared -including purple sulfur bacteria and sulfur reducing bacteria- suggests the reestablishment of redox conditions and stability of the wet patches. This “elastic” property of the community is also supported by an overrepresentation of anaerobic cycles within these wet patches. Although there were shifts in the community composition, the retention of functionality indicates a shared function in response to water reestablishment toward a normal condition within the pond. More interesting is the finding that in Lagunita under stress or under recovery, there is a bacterial core that is constant, meaning a set of shared RB persist over time.

Although the three sites are very close in space, the fact that they have different dynamics and diversity along the time-series indicates site specific community dynamics. This is not surprising given the large microbial beta-diversity observed in CCB in general (Bonilla-Rosso et al., 2012; Espinosa-Asuar et al., 2015; Pajares et al., 2016) and in the studied Lagunita pond in particular (Lee et al., 2017). We believe that such community differentiation in the space could be due to historical factors –such as early colonizers establishing the ground rules of interactions-, but also by stochastic events such as virus predation of the dominant taxa following a “king of the hill” model (Taboada et al., 2018).

The changes in diversity and function of site A, in particular, confirm our hypothesis that water conditions in Lagunita and at the whole Churince Lagoon are important factors influencing metabolic function-composition within microbial mats. Even though the lack of water constitutes an obvious environmental filter for aquatic microbes (Pontarp et al., 2012; Monard et al., 2016), the fluctuation of such an important resource seems to be playing a critical role in the distribution and abundance of the taxa shaping microbial assemblies within the mats.

### Microbial Mat Network Analysis

Modeling of interactions using networks is considered a powerful tool to understand the dynamics of succession within communities through time, as well as to analyze the stability within a system (Thébault and Fontaine, 2010; Coyte et al., 2015; Delmas et al., 2018). Unlike previous studies in which only the top 25% of interactions were used in the analysis (i.e., Weng et al., 2017), we used 100% of interactions, to gain information on the relationships among taxa given the importance of the RB in the diversity and function of the microbial mats of Lagunita.

In order to obtain the general patterns, we used the consensus network relationships and dissected them into positive and negative to find meaningful statistical properties. For instance, the network density or degree of network connectivity gives us an idea of how quickly perturbations may spread, by providing a measure of how dense the network is. A small diameter indicates presence of a densely connected nodes or hubs hence fast propagation among nodes, which may make the network more sensitive to perturbation (Delmas et al., 2018). The large network density obtained indicates that in Lagunita microbial mats, the networks of bacterial community are composed of highly connected groups. This was expected given the resilience of the system since a largely connected community is more robust to changes than low density networks (Sun et al., 2013).

It has been suggested that communities with modular organization of the type “small world” are more stable facing perturbations; modular arrangement allows different groups of nodes to perform different functions with some degree of independence (Newman, 2006). In practice, modularity values for biological networks typically fall in the range from about 0.3 to 0.7, and higher values are rare (Newman and Girvan, 2003). However, more recently, it has been suggested that it is not likely that the modularity maximum values (closer to one) always correspond to the best network for a stable community structure (Fortunato and Hric, 2016). Our study highlights the possibility that complex, densely connected networks can have modularity values that are lower than what it has been previously suggested (Newman and Girvan, 2003; Newman, 2006; Blondel et al., 2008; Poisot, 2013; Fortunato and Hric, 2016). In our study, the three microbial mat sampling sites are uniquely cohesive despite their large diversity. We consider that at CCB, the RB has been building complex bacterial communities that can survive under extremely unbalanced C: N: P conditions for a very long time (Souza et al., 2012) explaining the singularity of this site (Souza et al., 2018).

### Building Blocks of Microbial Mats Networks

Although the networks described herein are highly dense and similar to those obtained from random networks, the appearance of each network motif do not occur randomly. Rather, their presence across the TS-ENs in all taxonomic levels increase the robustness of the analysis. Therefore, evaluating such motifs provides a link to understand the unique type of relationship dynamics in contrasting sampling points.

Recently these motifs have been used to define species trophic roles in the context of their community and therefore, the network’s stability (Borrelli et al., 2015; Delmas et al., 2018). As far as we know, this is the first study to incorporate network motifs for the analysis of microbial mats under perturbation conditions. Further studies are needed to corroborate whether the overrepresentation of network motifs is specific to microbial mats or other environments, compared for example with neuronal, transcription and food webs (Tran et al., 2013; Borrelli et al., 2015).

Under the hypothesis than the environment can influence the strength and type of interactions among taxa, we expected to find similar interaction patterns among wet patches (sites B and C) while the dry patch at time 1 would be unique. Unexpectedly, site B was the only site that displayed a specific type of interaction, motif 74, a motif whose arrows suggest cross feeding among two members of the interaction, and a type of Black Queen dynamics toward the third member (Morris et al., 2012), suggesting a very unique mutualistic dynamic. In this site (B) we detected a wider range of interactions at the family level. Since all of these relationships are part of the microbial market and are context dependent (Mccully et al., 2017), we do not know what made site B unique. One possibility is that each site has a particular source of deep water by microfiltration. This idea may explain the specific RB dynamics within each site, since each one is “fed” from a slightly different seed bank.

To better understand why some motifs are found in high or low abundances within our microbial mats we need to explore not only the mathematical properties of such networks motif but also design experiments of direct interactions to understand the ecological meaning of generalists and specialists within each node.

### Keystone Taxa Are Part of the Rare Biosphere and of the Microbial Mat Core

In general, we found that site B has a higher number of hubs in max out-degree (four in total); meanwhile site C has three and the site A has only one hub. This suggests that the dry patch is possibly more fragile because if a single hub is removed, more relationships may be lost compared to sites B and C (see Table 3). In addition, we observed that most hubs belonging either to the RB or microbial mat core seem to provide PG. This is particularly meaningful, given that in ecological studies there is no way to discriminate the role of particular taxa within natural systems by simply highlighting their low abundance. However, in our study we found a direct implication of the RB in microbial mats under environmental perturbation.

We also observed that some of the hubs, which were detected independently in the three sites, were also detected in the global network. Interestingly, unclassified members of Eukarya domain were identified as a hub with positive relationships. It has been observed that diverse communities of eukaryotes live in microbial mats including not only a broad range of taxa, but also a large functional diversity, including phototrophs from several algal phyla and a variety of heterotrophic organisms. In this context, microbial mats could provide different microhabitats under contrasting conditions, which gives protection against oxidative, osmotic, freeze-thaw, and dehydration stressors for all microorganisms embedded within the microbial mat matrix (Jungblut et al., 2012). A global positive interconnection among unknown eukaryotic taxa within the global network in microbial mats of Churince is important to highlight regarding the presence of saprophytic, phagotrophic, parasitic, and predatory eukaryotes that would increase the number of links within a mat for nutrient and energy transfer (Duffy and Stachowicz, 2006). It would be very interesting to test these ideas by experimentally removing certain taxa (using particular antibiotics, for instance) and observing if their disappearance affects the entire system.

### Suggesting Possible Drivers of Microbial Mat’s Stability

Our results are in agreement with other studies that have proposed negative interactions increase the resilience of microbial communities (Foster and Bell, 2012; Coyte et al., 2015; Zelezniak et al., 2015; Deng et al., 2016). In addition, it has been suggested that microbial cooperative networks (characterized by mutualism) are often unstable, while a higher proportion of competitive interaction pairs (-/-) help the host maintain a stable microbial community in the case of the human microbiome (Coyte et al., 2015). However, it is expected that when resources are limited (as is the case of our extremely oligotrophic system), some species may outcompete others and stability is reached with one species per resource in the classic niche model (Borrelli et al., 2015). On the other hand, *in silico* studies suggested that modularity was able to have a positive effect on stability only when (a) the system is composed of two subsystems of about the same size and (b) the overall mean interaction strength is negative (Grilli et al., 2016). Here, consistent with the theory, we observed that in the stressed mat of site A the negative interactions are, on average, larger than the positive ones even though this site is the most resistant according to our ecological indexes.

However, under wet conditions there is an equilibrium between positive and negative interactions due to the metabolic interdependency based on cooperation or mutualistic relationships. This has been observed under nutrient-poor conditions where metabolic complementarity can provide group advantage (Morris et al., 2013). However, if this frail balance is tipped over by environmental disturbance, then the negative relationships exceed the positive ones. This type of behavior has been found in complex food webs due to low transformation of prey into predator (Allesina et al., 2015; Grilli et al., 2016). The latter do not apply for the CCB microbial mats since predators are virus and recycling of the nutrients are so efficient that we observe extremely low organic P (Lee et al., 2015). Furthermore, it has been suggested that large systems in which the positive interactions dominate, the negative ones are unstable and will likely lose stability through a “hop bifurcation” (conversion efficiency of resources of consumers) (Allesina et al., 2015). Hop bifurcation should be most common in the presence of an inverted biomass pyramid, typically occurring in planktonic or other aquatic systems (Allesina et al., 2015; Grilli et al., 2016). Moreover, it has been suggested that a strong network of interactions among organisms can provide a buffer against disturbance beyond the effect of functional redundancy, as alternative pathways (with different combinations of microbes) can be recruited to fulfill specific functions, thus increasing the negative interactions (Konopka et al., 2015). This is what we observed in our study: during wet conditions, the ratio of cooperation versus competition under equilibrium, however, negative interactions increase under dry conditions (see Table 3).

The hypothesis that closely coordinated metabolic associations promote homeostasis and become a buffer against stressful, resource-limited conditions has been previously described (Guerrero et al., 2002; Konopka et al., 2015; Wong et al., 2017). In general, is accepted that there is a metabolic and ecological congruence in a community as long as biogeochemical and environmental gradients allow individual niches to exist in close proximity. Thus, metabolically diverse microorganisms are oriented according to energetic, nutrient and ecological requirements and tolerances (Guerrero et al., 2002; Wong et al., 2017).

Metabolic dependencies based on the Black Queen hypothesis (Morris et al., 2012) are a starting point for the evolution of cooperative behavior, where the cross-feeding (bidirectional dependency) is obligated in communities were essential functions are costly for producers (Mas et al., 2016). To explore Black Queen ideas, we separated the effect of network complexity from specific traits of individual members in hubs. To find keystone candidate taxa important for the maintenance of structure and function of a community, we focused on microorganisms from the microbial mat core (present in all samples) and low abundant taxa (RB) to infer putative ecological niches and functional roles. We found that the majority of nodes are members of the RB and microbial mat core and despite their low abundance, their role in the community seems to be critical by establishing indirect, mutualistic relationships. An example of this is the case of a positive relationship between unclassified members of Oscillatoriales (cyanobacteria) and a sulfate reducing bacteria Desulfobacteraceae, along with a candidatus Parcubacteria and Rhodobacteraceae (heterotroph). In this example (see Figure 7C), the cyanobacteria release public goods in the form of carbon sources that are degraded by the candidatus Parcubacteria (Nelson and Stegen, 2015), making them available to Rhodobacteraceae. Therefore, by establishing mutualistic relationships, the RB is allowing co-occurrence of several small niches (Thébault and Fontaine, 2010; Zhou et al., 2010; Faust and Raes, 2012b; Mccully et al., 2017).

As aforementioned, in microbial mats the biogeochemical cycles through networks of metabolite exchange are structured along energetic gradients (Guerrero et al., 2002; Wong et al., 2017). As energy yields become limiting, these networks promote co-metabolic interactions to maximize energy disequilibrium (Wong et al., 2017). Thus, when there are more species than resources, some of them will invariably outcompete with others, in theory resulting in a final community with at most one species per resource, that should reach equilibrium and stability despite variable environmental parameter values (Borrelli et al., 2015).

## Conclusion

Microbial mats from three close by sites within Lagunita in the Churince system of the CCB displayed a high microbial diversity. Most of this diversity is represented by members of the RB, but also included a particular core of microorganisms that were present in all samples across spatial-temporal scales. Our analysis shows that when the aquifer re-established its deep flow, the anaerobic-dependent functions within the sulfur and methane cycles also reestablish. This rebound was likely possible due to a large “seed bank” that makes the microbial mat redundant and diverse. In their interaction motifs, we detected a type of site-specific “fingerprint,” even though they are few meters apart. The microbial mat under stressful conditions displayed more negative interactions than the wetter communities where mutualistic interaction balances with antagonism. This suggests an important role of the members of the RB in the permanence of these bacterial communities.

In conclusion, we suggest that the mechanisms behind microbial mats stability in Lagunita are related to an increase in negative interaction during perturbation, low modularity, large taxonomic diversity (represented by a large number of rare taxa), and core microorganisms which can carry out essential functions in the community. We consider that at CCB, the RB has been building complex bacterial communities that can survive under extremely unbalanced C: N: P conditions for a very long time explaining the singularity and resilience of this site, and we hope that this biodiversity will allow this wetland to be reborn from its stored seed bank.

## Data Availability Statement

The data and scripts to reproduce all the figures for this study can be found in the following repository https://valdeanda.github.io/Time_series_mats/. The software developed for the network analysis is found at https://valdeanda.github.io/NetAn/.

## Author Contributions

VDA, IZ-P, LE, and VS conceived the project and worked on the manuscript. VDA and JB performed the fieldwork, sampling, sample processing, DNA extraction and ecological indexes analysis. VDA, AP-H, and BC-M conducted the bioinformatic metagenomic analysis. BC-M and MH-R provided computing resources and analysis interpretation. MG-L, MH-R, and VDA designed and implemented NetAn. IZ-P, MG-L, NG-T, MH-R, and VDA provided ecological interpretation of the network analysis. LE and VS contributed to sampling and fieldwork, provided expertise, logistics and resources to develop and supervised the project, as well as intellectual contributions to the work. All authors made contributions to the manuscript.

## Conflict of Interest Statement

The authors declare that the research was conducted in the absence of any commercial or financial relationships that could be construed as a potential conflict of interest.

## Footnotes

1 http://bioinformatics.psb.ugent.be/webtools/Venn/

2 https://github.com/eead-csic-compbio/metagenome_Pfam_score/blob/master/config/config.txt

3 https://github.com/valdeanda/NetAn
