# Supplementary material for: Understanding the Mechanisms Behind the Response to Environmental Perturbation in Microbial Mats: A Metagenomic-Network Based Approach
Source: Front Microbiol. 2018 Nov 28;9:2606. doi: 10.3389/fmicb.2018.02606 (PMC6280815; doi:10.3389/fmicb.2018.02606)
Supplement: Supplementary file 1 [file Table_1.docx]

| **Supplementary Table 1. Physico-chemical characteristics of Lagunita pond during the study period of time** | | | | | |
| --- | --- | --- | --- | --- | --- |
| **Sampling time** | **Temperature (°C)** | **Conductivity (mS cm^-1^)** | **Salinity (ppm)** | **pH** | **Description Lagunita Pond** |
| Autumn (November 2012 ) | NR | NR | NR | NR | Disturbance |
| Spring (May 2013) | 20,27±0.25 | 8,32±0.03 | 4,70±0.01 | 8,60±0.01 | Humid |
| Autumn (October 2013) | 26.03±0.55 | 6.47±0.07 | 3.60±0.05 | 8.01±0.10 | Humid |
| Spring (May 2014) | 25,22±0.23 | 11,59±0.01 | 6,61±0.01 | 8,24±0.13 | Humid |
