# Supplementary material for: Understanding the Mechanisms Behind the Response to Environmental Perturbation in Microbial Mats: A Metagenomic-Network Based Approach
Source: Front Microbiol. 2018 Nov 28;9:2606. doi: 10.3389/fmicb.2018.02606 (PMC6280815; doi:10.3389/fmicb.2018.02606)
Supplement: Supplementary file 2 [file Table_2.docx]

| **Supplementary Table 2. Statistical comparison of metagenomic data** | | | | | | | |
| --- | --- | --- | --- | --- | --- | --- | --- |
| Sample | **Raw**  **reads x2**  **(PE-reads)** | **Trimming and Filtering**  **x2**  **(PE reads)** | **Assembly**  **(Megahit)** | | | | **Gene prediction**  **(Prodigal)** |
|  |  |  | **Contigs**  **(bp)** | **N50**  **(bp)** | **Max**  **(bp)** | **Avg. (bp)** | **Num of predicted proteins** |
| A1 | 1,658,827 | 1,375,081 | 342,065 | 403 | 39,849 | 408 | 360,384 |
| B1 | 847,223 | 592,721 | 100,016 | 516 | 31,440 | 536 | 123,738 |
| C1 | 2,032,826 | 1,475,508 | 332,018 | 453 | 66,899 | 459 | 375,848 |
| A2 | 2,170,214 | 1,755,043 | 439,801 | 415 | 9,060 | 426 | 480,122 |
| B2 | 2,447,533 | 2,085,860 | 364,150 | 415 | 25,075 | 426 | 402,287 |
| C2 | 1,633,385 | 1,376,563 | 385,873 | 403 | 7,939 | 402 | 408,069 |
| A3 | 2,378,839 | 2,041,813 | 473,101 | 402 | 8,176 | 407 | 491,897 |
| B3 | 1,763,139 | 1,501,213 | 465,425 | 409 | 19,838 | 417 | 508,107 |
| C3 | 2,714,75 | 2,285,940 | 482,822 | 400 | 36,798 | 415 | 515,946 |
| A4 | 2,027,738 | 1,660,009 | 387,179 | 396 | 5,513 | 395 | 406,115 |
| B4 | 2,193,111 | 1,906,342 | 391,814 | 400 | 5,786 | 404 | 420,130 |
| C4 | 3,012,348 | 2,497,262 | 521,665 | 403 | 11,783 | 408 | 557,927 |
| TOTAL | 22,165,183 | 20,553,355 | 4,685,929 |  |  |  | 5,050,570 |
