# Supplementary material for: Understanding the Mechanisms Behind the Response to Environmental Perturbation in Microbial Mats: A Metagenomic-Network Based Approach
Source: Front Microbiol. 2018 Nov 28;9:2606. doi: 10.3389/fmicb.2018.02606 (PMC6280815; doi:10.3389/fmicb.2018.02606)
Supplement: Supplementary file 6 [file Table_6.docx]

**Supplementary Table 6**. Score values obtained from MEBS algorithm. Asterisk represent those values above or equal FDR 0.0001. Red and green values indicate higher or lower scores respectively.

| **Name** | **ID-MGRAST** | **Sulfur** | **carbon** | **oxygen** | **iron** | **nitrogen** |
| --- | --- | --- | --- | --- | --- | --- |
| Alvinella pompejana –associated | 4441102.3 | 9.986 | 13.119 | 3.715 | 8.979 | 14.718 |
| PL mat | 4532771.3 | 9.718 | 24.142 | 8.074 | 8.385 | 21.366* |
| PL mat | 4532775.3 | 9.652 | 28.281 | 7.548 | 8.674 | 20.354* |
| PL mat | 4532772.3 | 9.574 | 21.964 | 6.732 | 8.754 | 19.872* |
| Activated sludge | 4493725.3 | 9.547 | 17.864 | 7.481 | 9.279 | 18.563 |
| C_Autumn_12 | C1 | 9.085 | 29.336 | 6.839 | 9.391 | 19.048* |
| B_Spring_13 | B2 | 8.989 | 35.881* | 6.839 | 9.391 | 19.226* |
| Phototrophic pink berry biofilm | 4454153.3 | 8.916 | 18.961 | 6.908 | 9.391 | 19.014 |
| A_Autumn_13 | A3 | 8.901 | 42.838* | 6.839 | 9.391 | 19.226* |
| C_Spring_14 | C4 | 8.901 | 37.170* | 6.839 | 9.391 | 19.226* |
| B_Autumn_12 | B1 | 8.831 | 36.355* | 6.135 | 9.339 | 18.829 |
| A_Spring_14 | A4 | 8.763 | 40.041* | 6.554 | 9.391 | 18.946 |
| B_Spring_14 | B4 | 8.763 | 40.041* | 6.554 | 9.391 | 18.826 |
| C_Autumn_13 | C3 | 8.714 | 35.156* | 6.853 | 9.391 | 19.18* |
| Shark Bay Postular mats | 4532716.3 | 8.704 | 36.203* | 5.902 | 9.391 | 18.866 |
| A_Spring_13 | A2 | 8.696 | 33.383 | 6.839 | 9.391 | 19.65* |
| Green mat CCB (reads) | 4441363.3 | 8.600 | 23.271 | 6.906 | 7.217 | 19.312* |
| Pavilon Lake mat | 532774.3 | 8.579 | 27.053 | 7.548 | 8.674 | 20.234* |
| B_Autumn_13 | B3 | 8.535 | 42.132* | 6.839 | 9.391 | 19.226* |
| A_Autumn_12 | A1 | 8.505 | 36.301* | 6.839 | 9.391 | 19.031* |
| C_Spring_13 | C2 | 8.262 | 33.394 | 6.839 | 9.391 | 18.785 |
| Polar mats | 4445126.3 | 8.261 | 21.365 | 7.686 | 8.010 | 18.871 |
| Sargasso Sea Water | 4441571.3 | 8.020 | 26.993 | 7.378 | 8.700 | 19.469* |
| Clinton Creek microbialite | 4532705.3 | 7.814 | 12.137 | 6.745 | 8.494 | 18.620 |
| Shark bay Smooth mat | 4532715.3 | 7.509 | 29.551 | 7.076 | 8.234 | 18.460 |
| AMD biofilm | 4441138.3 | 7.173 | 15.988 | 7.046 | 6.455 | 17.833 |
| Acidic Hot Spring Coquito | 4449206.3 | 5.856 | 14.253 | 7.160 | 3.957 | 16.082 |
| Hydrothermal vent Taiwan | 4487624.3 | 5.845 | 10.709 | 6.531 | 8.635 | 17.884 |
| Guerrero Negro microbial mat | 4440966.3 | 5.817 | 8.804 | 1.178 | 4.579 | 11.697 |
| Pavilon Lake microbial mat | 4526739.3 | 5.703 | 26.295 | 6.092 | 5.399 | 16.345 |
| Polar microbial mat | 4445129.3 | 5.672 | 16.691 | 7.727 | 8.394 | 18.842 |
| Red mat CCB(reads) | 4442466.3 | 5.359 | 13.446 | 7.593 | 7.638 | 18.965 |
| AMD biofilm | 4441137.3 | 5.159 | 9.276 | 7.634 | 4.824 | 17.251 |
| Clinton Creek sediment | 4532704.3 | 4.960 | 15.289 | 7.562 | 7.317 | 19.213* |
| Marine stromatolitic mat from Highborne Cay | 4449590.3 | 4.880 | 19.624 | 6.447 | 6.901 | 16.208 |
| Green Mat CCB (contigs) | 4441347.3 | 4.822 | 14.043 | 7.462 | 2.749 | 15.454 |
| Hydrothermal vent Taiwan | 4487625.3 | 4.760 | 12.707 | 5.715 | 9.282 | 17.467 |
| Pavilon Lake mat | 4526738.3 | 4.035 | 16.771 | 6.831 | 3.386 | 15.172 |
| Hot spring microbial mat Yellowstone | 4443746.3 | 3.698 | 9.184 | 7.268 | 3.146 | 11.424 |
| Hot spring microbial mat Yellowstone | 4443749.3 | 3.520 | 10.772 | 8.047 | 3.474 | 14.217 |
| Polar cryoconite | 4491734.3 | 3.306 | 14.757 | 6.176 | 6.404 | 16.589 |
| Pavilon Lake mat | 4526740.3 | 3.299 | 15.929 | 2.892 | 3.435 | 12.699 |
| Red mat CCB (reads) | 4442467.3 | 3.098 | 8.511 | 7.088 | 8.758 | 17.550 |
| Hot spring microbial mat Yellowstone | 4443750.3 | 2.660 | 3.959 | 7.052 | 3.709 | 10.587 |
| Stromatolite Pozas Azules II CCB | 4440067.3 | 2.514 | 15.546 | 7.409 | 5.817 | 15.775 |
| Hot spring microbial mat Yellowstone | 4443762.3 | 1.188 | 9.122 | 7.319 | 3.474 | 12.362 |
| Pavilon Lake microbial mat | 4532785.3 | 1.118 | 4.183 | 6.948 | 5.250 | 14.997 |
| Rio Mesquites Oncolite | 4440060.4 | 1.047 | 7.701 | 8.482 | 5.203 | 15.646 |
| CO2 reducing sediment Yellowstone | 4514452.3 | 0.676 | 0.924 | -0.357 | 1.338 | 2.576 |
| Hot spring microbial mat Yellowstone | 4443747.3 | 0.378 | 4.295 | 2.456 | 0.542 | 3.722 |
| Shark Bay c  olumnar stromatolite viral fraction | 4466753.3 | 0.24 | -0.247 | -0.065 | 0.356 | 0.713 |
| Stromatolite Bahamas | 4440061.3 | -0.246 | 0.597 | -0.011 | 0.54 | 1.467 |
| Pavilon Lake microbial mat | 4532783.3 | -1.191 | 0.486 | 3.600 | 4.373 | 10.322 |
| Pavilon Lake microbial mat | 4532784.3 | -1.264 | 1.615 | 6.409 | 6.466 | 12.889 |
| Grassland | 4511045.3 | -2.295 | 1.790 | 5.412 | 2.745 | 13.024 |
| Open Ocean Caribbean Sea Rosario Honduras | 4441588.3 | -2.367 | -1.331 | 0.944 | 1.173 | 1.801 |
| Open Ocean Panama | 4441592.3 | -2.584 | 0.205 | 2.314 | 4.147 | 7.948 |
| Soil | 4489656.3 | -2.649 | 0.596 | 3.933 | 3.329 | 10.872 |
