# Supplementary material for: Understanding the Mechanisms Behind the Response to Environmental Perturbation in Microbial Mats: A Metagenomic-Network Based Approach
Source: Front Microbiol. 2018 Nov 28;9:2606. doi: 10.3389/fmicb.2018.02606 (PMC6280815; doi:10.3389/fmicb.2018.02606)
Supplement: Supplementary file 7 [file Table_7.docx]

**Supplementary Table 7.** Statistics derived from Metamis simulator**.**

| Site | Level | N taxa | IN^a^ | BCD^d^ | HA^b^ | LA^c^ | Rare | Gap  (LA/nC) | Gap  (Rare) | Total Taxa | CN^e^ |
| --- | --- | --- | --- | --- | --- | --- | --- | --- | --- | --- | --- |
| A | Phylum | 100 | 78 | 0,081 | 8 | 10 | 67 | 1 | 1 | 85 | 61 |
|  | Class | 168 | 130 | 0,127 | 10 | 28 | 101 | 1 | 2 | 139 | 91 |
|  | Order | 302 | 144 | 0,14 | 22 | 52 | 182 | 1 | 2 | 255 | 207 |
|  | Family | 539 | 169 | 0,138 | 27 | 82 | 344 | 1 | 4 | 450 | 354 |
| B | Phylum | 100 | 86 | 0,134 | 7 | 12 | 73 | 1 | 1 | 92 | 68 |
|  | Class | 168 | 138 | 0,127 | 10 | 28 | 109 | 1 | 1 | 147 | 123 |
|  | Order | 302 | 154 | 0,14 | 17 | 55 | 196 | 1 | 2 | 267 | 219 |
|  | Family | 539 | 161 | 0,143 | 19 | 92 | 341 | 1 | 5 | 452 | 332 |
| C | Phylum | 100 | 86 | 0,137 | 8 | 10 | 75 | 1 | 1 | 93 | 69 |
|  | Classs | 168 | 146 | 0,14 | 8 | 28 | 117 | 1 | 1 | 153 | 129 |
|  | Order | 302 | 143 | 0,159 | 20 | 52 | 181 | 1 | 2 | 253 | 205 |
|  | Family | 539 | 165 | 0,17 | 21 | 92 | 364 | 1 | 5 | 474 | 354 |
|  | Total |  | 1600 |  |  |  |  |  |  |  |  |

^a^ Intermediate Networks; ^b^ High Abundance (>0.01), ^c^ Low Abundance (<0.001); ^d^ Bray-Curtis dissimilarity index; ^e^ CN Consensus network
