# Supplementary material for: Understanding the Mechanisms Behind the Response to Environmental Perturbation in Microbial Mats: A Metagenomic-Network Based Approach
Source: Front Microbiol. 2018 Nov 28;9:2606. doi: 10.3389/fmicb.2018.02606 (PMC6280815; doi:10.3389/fmicb.2018.02606)
Supplement: Supplementary file 8 [file Data_Sheet_1.docx]

***Supplementary Material***

**Understanding the mechanisms behind the response of environmental perturbation in microbial mats: a metagenomic-network approach**

***Valerie De Anda^1^, Icoquih Zapata-Peñasco ^2^, Jazmín Blaz^3^, Augusto Cesar Poot-Hernández^4^, Bruno Contreras Moreira^5,6^, Marcos González Laffitte^7^, Niza Gámez Tamariz^1^, Maribel Hernández Rosales^7,^ Luis E. Eguiarte^1^ and Valeria Souza****

*** Correspondence:** Corresponding Author: [*souza@unam.mx*](mailto:souza@unam.mx)

**
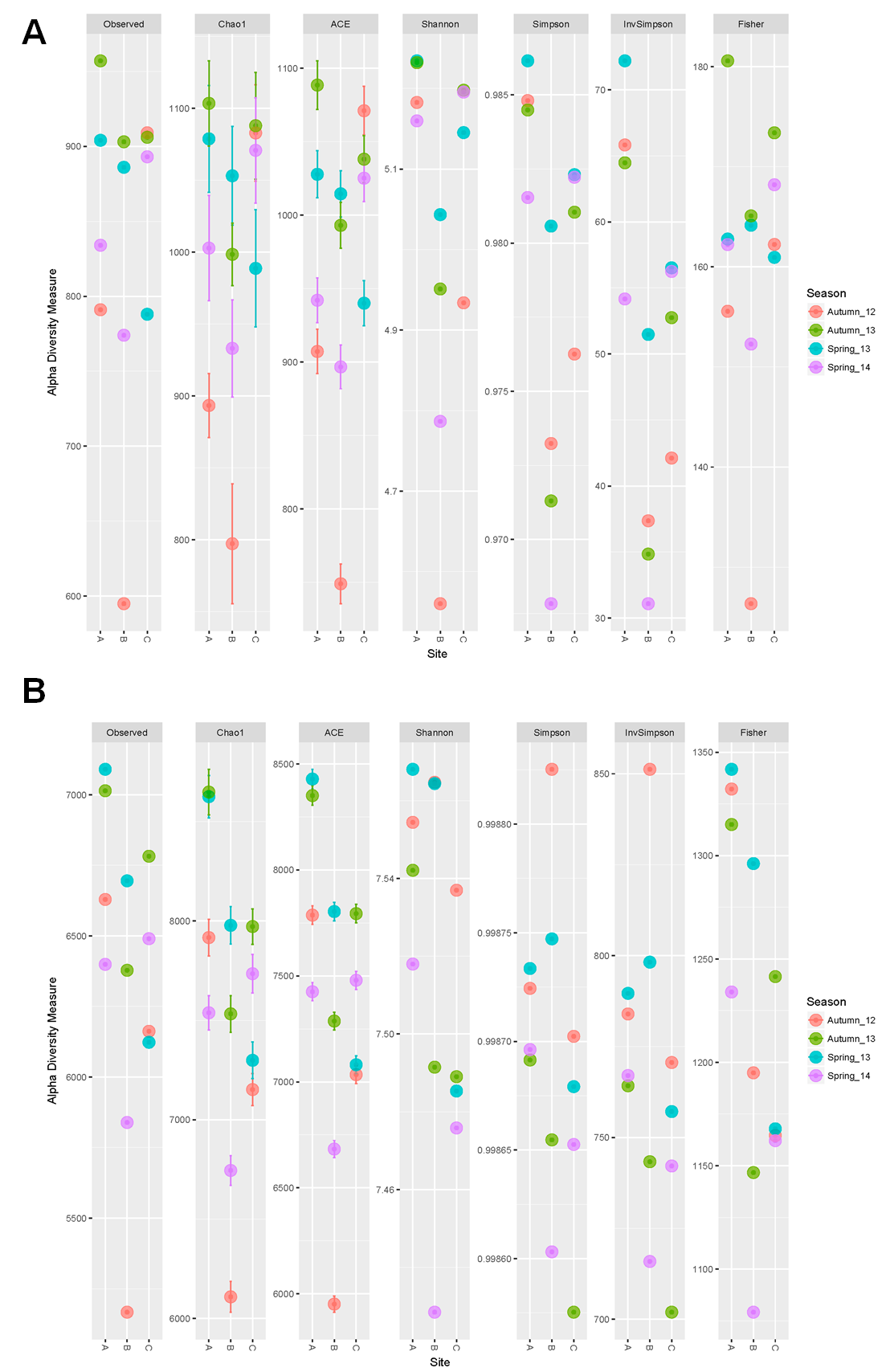
**

**Supplementary Figure 1**. Several estimators of Alpha-richness in the microbial mats samples at taxonomic (panel A), and metabolic level (panel B).

**
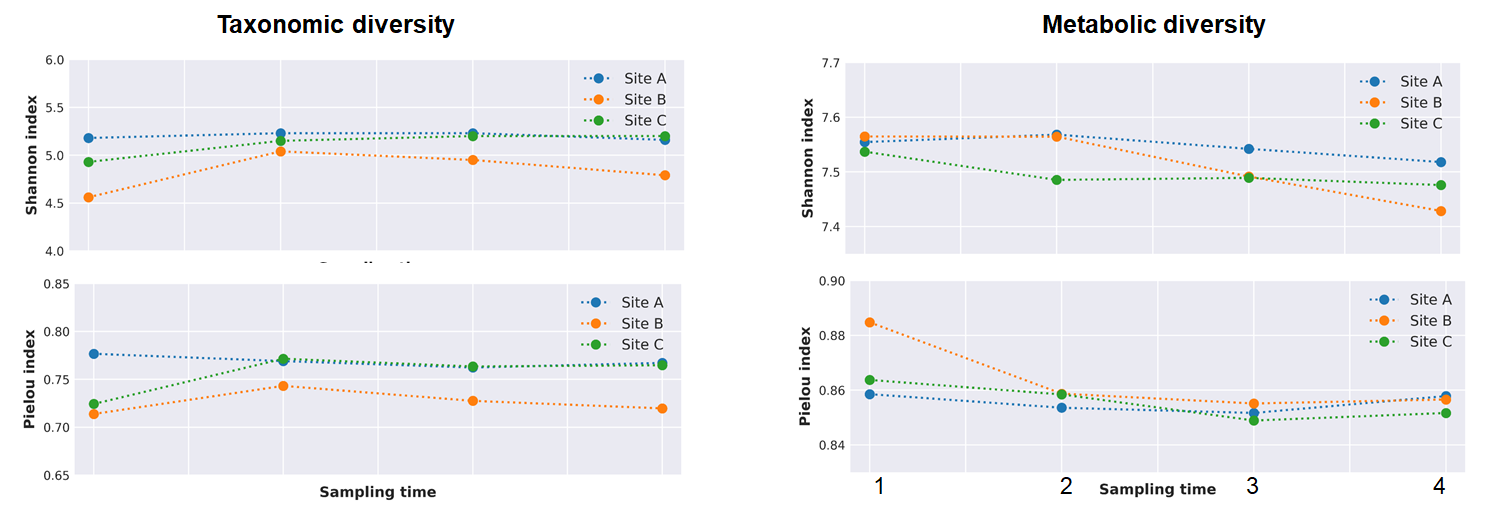
Supplementary Figure 2.** Variation through time of Shannon and Pielou diversity across sites at taxonomic (A) and metabolic (B), level.

**
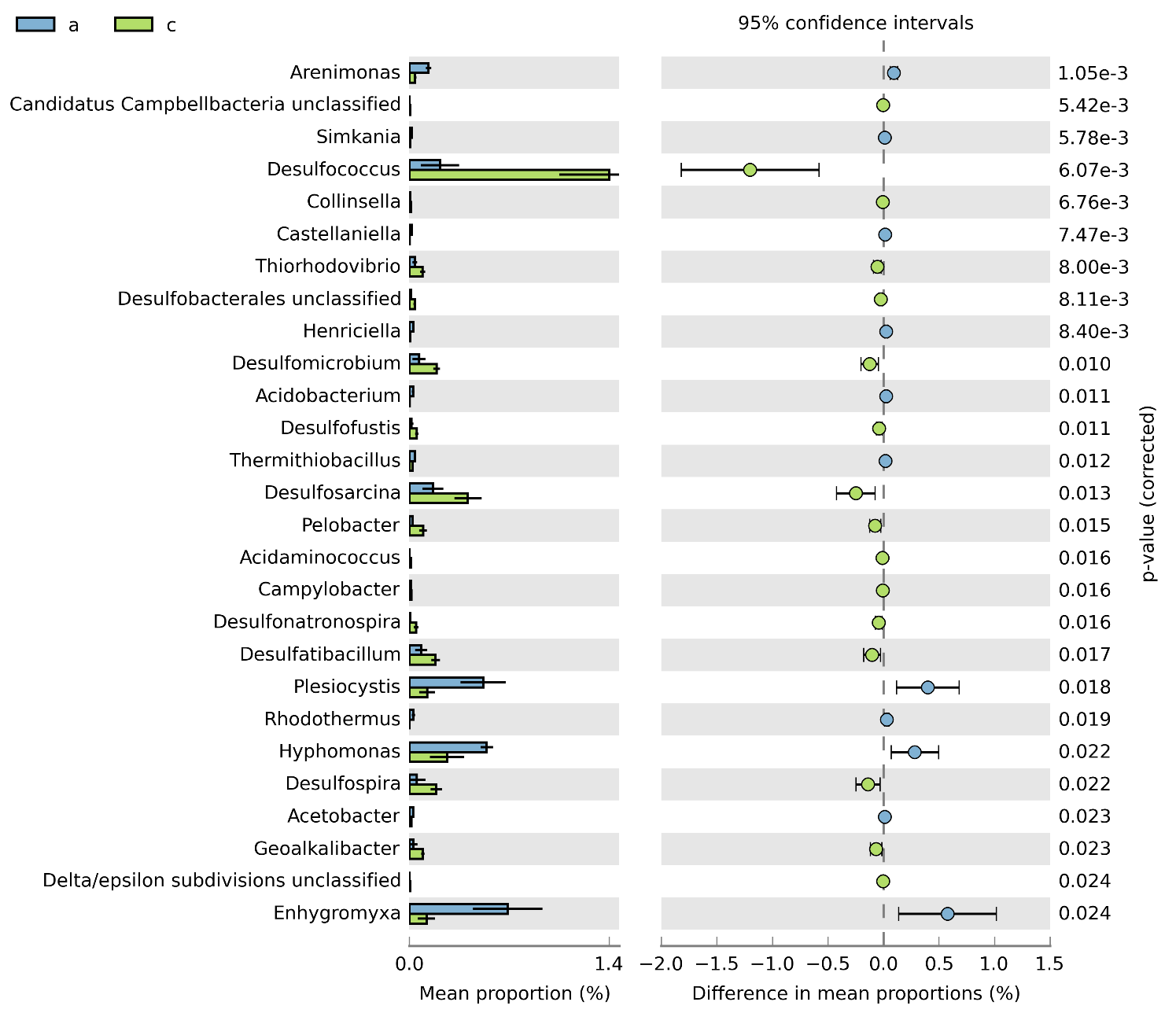
**

**Supplementary Figure 3.**  Extender error bar indicating all genera where Welch’s t-test with confidence interval method DP welch’ inverted of 0.95 produces a p-value (>0.025). The difference in mean proportion between the microbial mats from site A and C are shown in blue and green colors respectively.

**
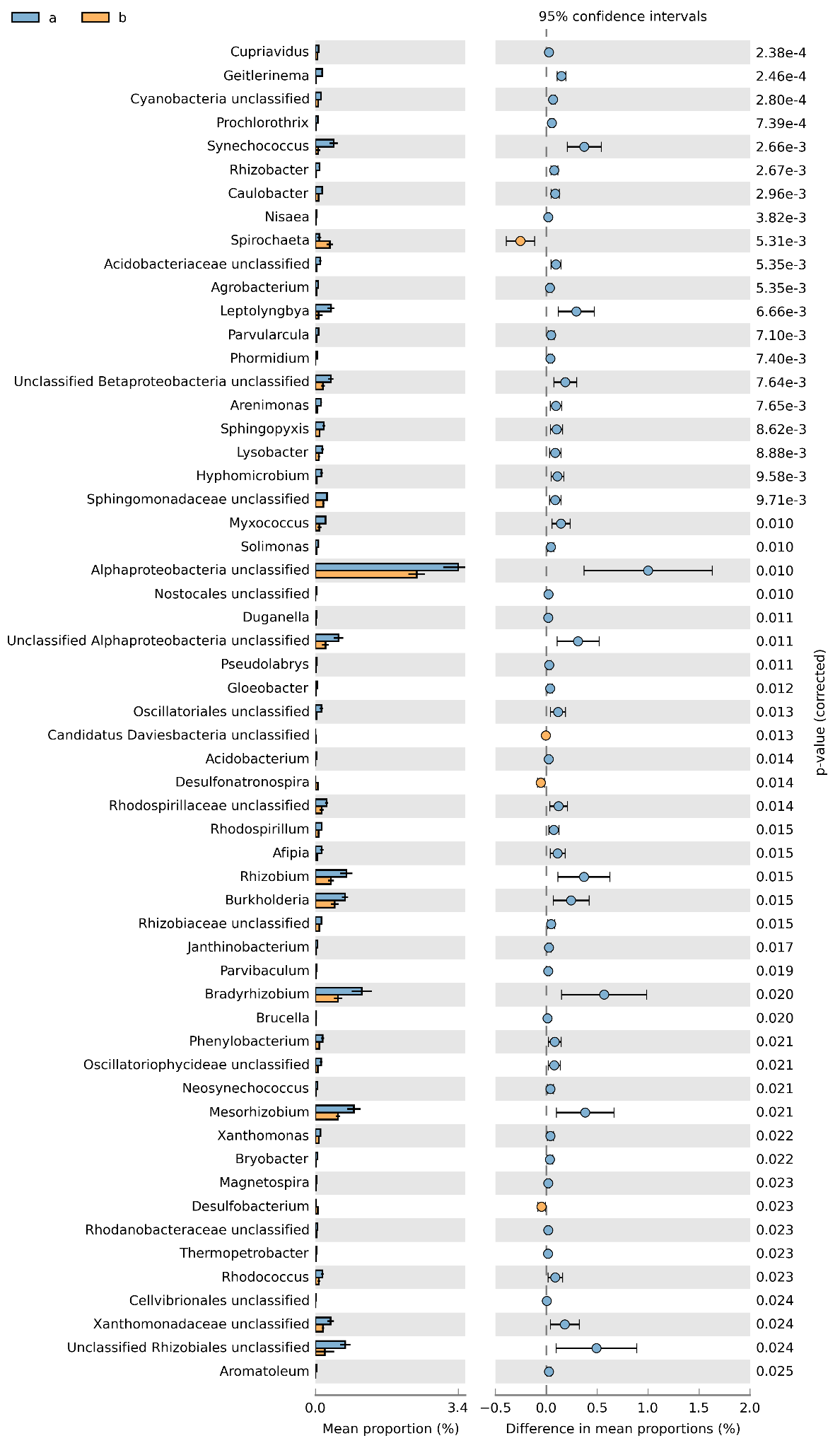
**

**Supplementary figure 4.**  Extender error bar indicating all genera where Welch’s t-test with confidence interval method DP welch’ inverted of 0.95 produces a p-value (>0.025). The difference in mean proportion between the microbial mats from site A and B are shown in blue and orange colors respectively.

**
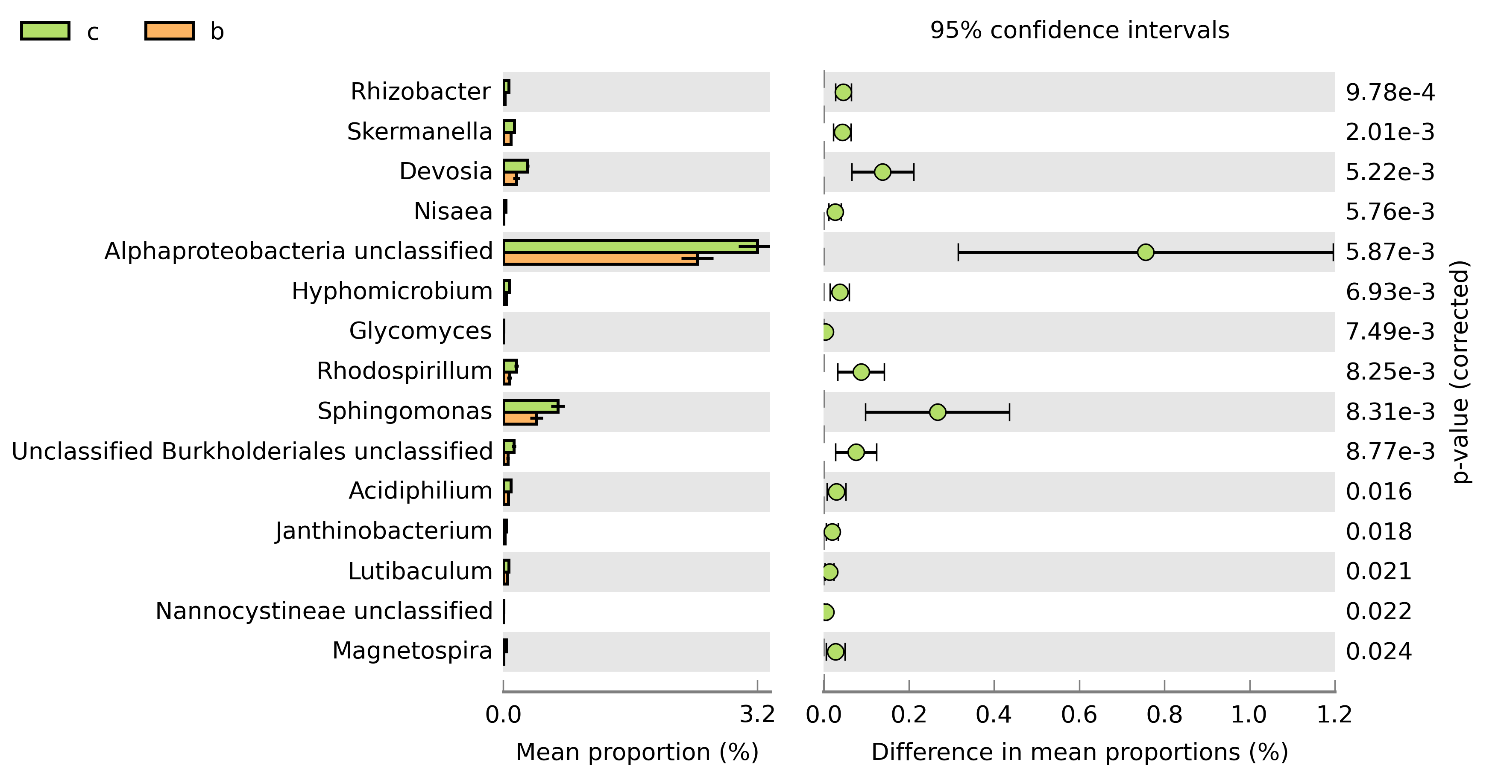
**

**Supplementary figure 5.**  Extender error bar indicating all genera where Welch’s t-test with confidence interval method DP welch’ inverted of 0.95 produces a p-value (>0.025). The difference in mean proportion between the microbial mats from site B and C are shown in orange and green colors respectively.
